# A Disorder-Aware Computational Framework to Identify Structurally Tractable Targets in Proliferative Vitreoretinopathy

**DOI:** 10.1016/j.xops.2026.101249

**Published:** 2026-05-25

**Authors:** Mak B. Djulbegovic, Nedym Hadzijahic, David J. Taylor Gonzalez, Michael Antonietti, Sidra Zafar, Ajay E. Kuriyan

**Affiliations:** 1 Wills Eye Hospital, Thomas Jefferson University Hospital, Philadelphia, Pennsylvania; 2 University of Miami, Miami, Florida; 3 Department of Ophthalmology, Broward Health North, Pompano Beach, Florida; 4 Department of Ophthalmology, Massachusetts Eye and Ear Infirmary, Harvard Medical School, Boston, Massachusetts; 5 Mid Atlantic Retina at Wills Eye Hospital, Philadelphia, Pennsylvania

**Keywords:** Proliferative vitreoretinopathy, Epithelial–mesenchymal transition, Intrinsic disorder, SNAIL1, Artificial intelligence

## Abstract

**Objective:**

Proliferative vitreoretinopathy (PVR) remains a major cause of failure after rhegmatogenous retinal detachment repair and lacks effective pharmacologic therapies. Although epithelial–mesenchymal transition (EMT) is central to PVR pathogenesis, the structural determinants governing the tractability of EMT regulators, particularly those involving intrinsic disorder, remain poorly defined. We developed a disorder-aware, artificial intelligence–enabled computational framework to evaluate EMT-associated proteins in PVR and prioritize structurally tractable regulators for structure-based targeting.

**Design:**

A computational, hypothesis-generating study employing an in silico screening and structural modeling pipeline.

**Subjects:**

No human subjects or biological specimens were included. The dataset comprised 25 EMT-associated proteins implicated in PVR, curated through a narrative review of peer-reviewed literature.

**Methods:**

Candidate proteins were evaluated using a multistage pipeline integrating intrinsic disorder profiling (Rapid Intrinsic Disorder Analysis Online), redox-sensitive disorder-to-order transition (DOT) analysis (AIUPred), and protein–protein interaction network assessment (Search Tool for the Retrieval of Interacting Genes/Proteins [STRING]). Structure-based modeling and generative binder design were then applied to the top-ranked candidate using RFdiffusion for de novo backbone generation, protein message passing neural network for sequence design, and AlphaFold2 for structural validation.

**Main Outcome Measures:**

Primary measures were the proportion of intrinsically disordered residues, redox-sensitive disorder change, STRING network coherence within EMT-related pathways, and the structural consistency of the designed binder–target complex, assessed by root mean square deviation (RMSD) and mean per-residue confidence (predicted local distance difference test [pLDDT]).

**Results:**

Of the 25 EMT-associated proteins screened, several exhibited intermediate intrinsic disorder profiles and measurable DOT potential. Snail Family Transcriptional Repressor 1 (SNAIL1) emerged as the highest-priority candidate, demonstrating an intermediate intrinsic disorder profile (∼35%), a pronounced redox-sensitive DOT region, and selective connectivity within EMT-related signaling networks. Functional mapping of the SNAIL1 C-terminal DOT segment identified 6 basic residues with literature-supported or motif-based regulatory significance (K187, R191, R224, K234, K253, and R264). Following sequence design and structural validation, the top-ranked binder exhibited the lowest structural deviation within the generated ensemble (RMSD 18.5 Å) and high per-residue confidence (mean pLDDT 0.84).

**Conclusions:**

Our study introduces a disorder-informed computational framework for prioritizing structurally tractable EMT regulators in PVR. As a proof-of-concept, the pipeline nominates SNAIL1 and generates a structure-aware de novo binder targeting its C-terminal DOT region, providing a foundation for disorder-based therapeutic discovery in fibrotic retinal disease.

**Financial Disclosure(s):**

Proprietary or commercial disclosure may be found in the Footnotes and Disclosures at the end of this article.

Proliferative vitreoretinopathy (PVR) remains one of the most severe complications of retinal detachment (RD) repair surgery. It is characterized by fibrocellular membrane proliferation on either the retinal surface or within the retina, resulting in tractional forces often leading to a recurrent RD and suboptimal visual outcomes.^1^, ^2^, ^3^ Despite advances in vitreoretinal surgical techniques, PVR remains notoriously difficult to manage and is a leading cause of failure in rhegmatogenous RD repair, with the resulting tractional detachment often leading to suboptimal visual recovery.^2^^,^^4^ Epidemiological estimates suggest that 5% to 10% of patients with primary rhegmatogenous RD will develop PVR, though these rates may be higher in cases involving giant retinal tears, choroidal detachment, ocular trauma, or uveitis.^5^, ^6^, ^7^ When recurrent detachments occur, patients face an escalating cycle of surgical interventions, including repeated vitrectomies and retinectomies, which is often associated with a reduced visual prognosis.^8^^,^^9^ The current mainstay of treatment is surgical, typically involving pars plana vitrectomy with membrane peeling and retinal reattachment, sometimes combined with scleral buckling or retinectomy.^2^^,^^3^^,^^10^ Adjunctive pharmacologic agents, such as methotrexate, corticosteroids, and 5-fluorouracil, have been explored, but none have achieved widespread clinical adoption due to limited and heterogeneous evidence.^2^^,^^11^^,^^12^ There are no Food and Drug Administration-approved treatments for PVR, despite extensive research into anti-inflammatory, antiproliferative, and antifibrotic strategies. Given these relatively high rates and the persistent lack of effective medical therapies, our study seeks to address this gap by investigating the molecular basis of PVR.

While multiple mechanisms have been proposed to drive PVR progression, including inflammation, oxidative stress, and abnormal wound healing, epithelial–mesenchymal transition (EMT) remains the prevailing hypothesis.^1^ Experimental wet-lab studies have shown that inhibition of EMT can attenuate fibrotic progression in PVR.^13^, ^14^, ^15^ From a mechanistic standpoint, EMT involves the transformation of retinal pigment epithelial (RPE) cells into contractile, myofibroblast-like cells, accompanied by loss of cell polarity and intercellular adhesion, contributing to fibrotic membrane formation and tractional RD.^16^, ^17^, ^18^ This process is regulated by multiple signaling pathways, including transforming growth factor beta (TGF-β)/Smad, ERK1/2, Rho, mitogen-activated protein kinase, Notch, and Wnt/β-catenin.^19^^,^^20^ In addition, various proteins participate in forming and sustaining these fibrotic membranes. For instance, TSPAN4-positive migrasomes released by RPE cells can initiate pathogenic cascades, and single-cell analyses of epiretinal membranes have revealed widespread molecular deregulation.^21^^,^^22^ Yes-associated protein (YAP) is also implicated in PVR progression through its regulation of EMT in the retina, while epithelial membrane protein-2 contributes to fibrotic membrane formation.^23^^,^^24^ Notably, Snail Family Transcriptional Repressor 1 (SNAIL1), a zinc-finger transcription factor and key EMT regulator, has been shown to induce EMT in RPE cells, triggering morphological changes directly, downregulation of epithelial markers (E-cadherin, ZO-1), and upregulation of mesenchymal markers (alpha–smooth muscle actin and fibronectin), ultimately enhancing cell motility.^18^^,^^25^^,^^26^ The identification of these diverse targets underscores the crucial role that proteins play in the pathogenesis of PVR.

Beyond traditional structure–function paradigms, structural plasticity and intrinsically disordered proteins (IDPs) have emerged as critical drivers of cellular transformation in various ocular pathologies. This is evident in ocular surface squamous neoplasia, where mutations in titin, a giant protein with abundant intrinsically disordered regions, promote chromosomal instability and confer resistance to interferon therapy.^27^ Similarly, in conjunctival melanoma, driver mutations in genes such as BRAF and PTEN frequently localize to regions of high intrinsic disorder, likely perturbing critical protein–protein interaction (PPI) networks and driving tumor progression.^28^ Similarly, the metastatic biomarker preferentially expressed antigen in melanoma utilizes a disorder-to-order transition (DOT) region near its C-terminus to facilitate functional interactions in class 1 uveal melanoma.^29^ Furthermore, structural plasticity in the PROS1/MERTK signaling axis has been implicated in driving neoplastic development through enhanced conformational adaptability. These findings from ocular oncology suggest that similar protein-level dynamism may govern the EMT and fibrotic membrane formation characteristic of PVR.

This raises an important and underexplored question: What role might structural plasticity, specifically intrinsic disorder, play in these proteins? A growing body of literature highlights how IDPs challenge traditional structure–function paradigms, instead functioning through dynamic ensembles and conditional folding.^30^, ^31^, ^32^ Notably, IDPs often harbor short linear motifs or molecular recognition features that become functionally active upon DOTs, an event that can be critical in driving pathological processes.^32^, ^33^, ^34^ Despite their recognized importance in other diseases, the role of intrinsic disorder in PVR remains largely unexplored. Our group is particularly interested in exploring de novo protein design to target such disordered regions, given that no current approaches fully address this molecular dimension in PVR. We propose that the integration of artificial intelligence (AI)-driven modeling with an understanding of protein disorder offers a novel framework for interrogating and, if appropriate, therapeutically modulating PPI networks central to PVR pathogenesis.

In 2024, the exponential growth of AI in structural biology has opened new frontiers for investigating such molecular intricacies. This paradigm shift was underscored by a recent Nobel Prize in Chemistry, which recognized breakthroughs in protein structure prediction and de novo protein design that enabled the development of tools such as AlphaFold and RFdiffusion.^35^^,^^36^ Deep learning–based platforms, including AlphaFold, RosettaFold, and Evolutionary Scale Modeling-Fold, can now predict protein structures at near-atomic resolution, even for challenging targets such as those with large intrinsically disordered regions.^37^, ^38^, ^39^ These advances improve our understanding of structure and enable the identification of previously inaccessible druggable sites, particularly in disordered or partially structured regions.

Building on this progress, our study introduces a multistage computational pipeline designed to identify and evaluate EMT-associated proteins in PVR, to nominate and structurally characterize the most attractive disorder-informed drug targets. The approach integrates a literature review, sequence-based disorder prediction, structural modeling, and generative design to prioritize proteins based on predicted disorder, druggability features, and suitability for structure-based targeting. This framework enables rational ranking of candidate proteins, particularly those with disordered regions that may mediate transient molecular interactions and represent novel therapeutic interfaces. To demonstrate its feasibility, we apply this pipeline to a high-priority candidate emerging from our evaluation and use RFdiffusion to design hypothetical binding motifs that could stabilize DOT regions, offering a potential route toward therapeutic modulation of fibrotic signaling in PVR.

## Methods

### Identification of a Target Protein

#### Literature Review

To identify proteins involved in EMT in PVR, we performed a narrative review of peer-reviewed literature and major scientific databases. The objective of the literature review was to curate a list of EMT-related proteins implicated in PVR, which were then assessed using computational tools to evaluate intrinsic disorder, structural characteristics, and druggability potential.

#### Protein Selection and FASTA Sequence Acquisition

Following the identification of key EMT-related proteins from the literature review, we obtained each protein’s canonical primary amino acid sequences from the Universal Protein Resource (UniProtKB) (https://www.uniprot.org/).^40^ Using the identified gene names and protein targets, we used the UniProt ID mapping tool to retrieve a list of unique UniProt accession numbers, ensuring that only *Homo sapiens* sequences were selected. From these unique UniProt IDs, FASTA sequences were extracted and formatted for downstream computational analysis.

#### Druggability Assessment

To identify potential disorder-based drug targets in PVR, we developed a computational druggability assessment framework integrating intrinsic disorder content, DOTs, and functional relevance in EMT. Canonical FASTA sequences for each protein identified in our literature review (see Literature Review) were obtained from UniProtKB (Fig S1, available at www.ophthalmologyscience.org). Intrinsic disorder profiles were assessed using the Rapid Intrinsic Disorder Analysis Online (RIDAO) platform (https://RIDAO.app; accessed January 31, 2025), which integrates multiple per-residue disorder predictors and aggregate outputs, including PONDR VLXT, PONDR VSL2B, PONDR VL3, IUPred-Short, IUPred-Long, PONDR-FIT, and the mean disorder profile.^41^ Proteins displaying intrinsic disorder values between 30% and 80% were considered within the acceptable range for druggability, balancing flexibility with partial order. This intermediate disorder range is enriched for conditionally structured regions that retain sufficient conformational plasticity to mediate transient, regulatory PPIs, while still forming locally ordered interfaces capable of stabilizing upon binding or DOT. In contrast, proteins at the extremes of disorder either lack the structural adaptability required for inducible binding (<20%) or fail to present persistent, targetable interaction surfaces (>85%), limiting their suitability for structure-informed therapeutic design. Proteins with low disorder (<20%) or extreme disorder (>85%) were excluded from further analysis.

To identify DOT regions, we used AIUPred v2 (https://iupred.elte.hu; accessed January 31, 2025) in its redox-state prediction mode with default smoothing enabled.^42^ This module estimates intrinsic disorder under both redox-minus and redox-plus conditions. The degree of redox-dependent conformational change was quantified as Δ disorder, calculated as the difference between the mean disorder scores under redox-plus and redox-minus conditions (Δ disorder = redox-plus – redox-minus). Regions with Δ disorder ≥0.20 are considered to exhibit meaningful DOTs and reflect conformational adaptability and redox-sensitive flexibility. The redox-state mode was selected because oxidative stress is a recognized component of the PVR microenvironment and can influence EMT-associated signaling, making redox-sensitive conformational behavior a biologically relevant dimension for prioritizing DOT regions.^43^^,^^44^ We therefore used redox-dependent disorder profiling not to claim a specific in vivo oxidation state for each candidate protein, but as a comparative screening strategy to identify proteins with condition-responsive structural plasticity that may be more amenable to regulatory targeting.

Protein–protein interaction context was evaluated using Search Tool for the Retrieval of Interacting Genes/Proteins (STRING) v12.0 (https://string-db.org/; accessed January 31, 2025).^45^ Networks were generated using the full STRING network setting, evidence-based edges, and all active interaction sources available through the interface at the time of analysis (text mining, experiments, databases, co-expression, neighborhood, gene fusion, and co-occurrence). The minimum required interaction score was set to the highest confidence threshold (0.900), and the maximum number of interactors displayed was set to 500 in the first shell, with no second-shell expansion. Proteins were prioritized if they exhibited selective, biologically coherent connectivity within EMT-relevant pathways rather than broad hub-like interaction patterns.

### Evaluation of a Target Protein–SNAIL1

#### SNAIL1 Structural Assessment

SNAIL1 was selected as the high-priority target protein based on its central regulatory role in EMT, established pathogenic relevance in PVR, and intermediate intrinsic disorder profile identified through our computational assessment. To obtain a structural representation of the human SNAIL1 transcription factor, we used a predicted model generated by AlphaFold2 (DeepMind, EMBL-EBI).^35^ AlphaFold was selected because it remains among the current state-of-the-art deep learning platforms for high-accuracy protein structure prediction, integrating multiple sequence alignments and structural templates through an attention-based neural network architecture. While other frontier models exist, AlphaFold was chosen arbitrarily for this step, as our objective was not to compare modeling platforms but to obtain a representative structural representation of the SNAIL1 protein prior to downstream analysis. The predicted structure was retrieved from the AlphaFold Protein Structure Database (https://alphafold.ebi.ac.uk/entry/O95863) and used to qualitatively confirm the presence of structural features relevant to our disorder-based targeting strategy.

#### Functional Mapping of the SNAIL DOT Region

Following identification of the C-terminal DOT region of SNAIL1 (residues 181–264) in the preceding analyses (see section Druggability Assessment for more details on AIUPred analysis), literature-supported post-translational modification (PTM) and functional information was compiled from peer-reviewed sources and curated protein databases to annotate residues within this region with known or suspected regulatory roles, including acetylation, SUMOylation, ubiquitination, and nuclear localization or DNA-binding functions. Each position within the 181 to 264 window was evaluated according to its documented or proposed involvement in these regulatory processes, with particular emphasis on positively charged residues in motifs previously implicated in SNAIL1 C-terminal function.

Residues that satisfied one or more annotation criteria were provisionally classified as candidate interface positions, representing potential sites of structural or regulatory significance. These candidate positions were subsequently used to define the interaction surface for binder design using the RFdiffusion framework described in section RFdiffusion-Based Binder Design.

#### RFdiffusion-Based Binder Design

As described in the previous section, using a combination of redox-sensitive DOT mapping, structural modeling of the zinc-finger domain, and literature-curated PTM/functional data, we prioritized 6 basic residues (K187, R191, R224, K234, K253, and R264) as a contiguous, interface-defining epitope within the DOT region. These residues were then used as the design target for RFdiffusion-based binder generation. Binder design was performed using the RFdiffusion pipeline implemented in a Google Colab notebook (available at: https://colab.research.google.com/github/sokrypton/ColabDesign/blob/main/rf/examples/diffusion_ori.ipynb). The target structure was based on SNAIL1, and a single 80-residue binder was designed against 6 identified residues as defined in the previous section: 187, 191, 224, 234, 253, and 264. A monomeric binder was generated (copies = 1) because the design objective involved a single-target interaction rather than an oligomeric assembly. The 80-residue length was selected to provide a compact, single-domain binder scaffold large enough to support a stable folded core and present a defined interaction surface, while remaining small enough to minimize unnecessary structural complexity and conformational heterogeneity during de novo design. This size is consistent with the general scale of many experimentally tractable mini-protein binders used in structure-guided design workflows.^36^

RFdiffusion was run for 200 iterations to permit adequate backbone refinement. Although symmetry was set to its default cyclic setting, this had no functional effect due to the monomeric design. Following backbone generation, protein message passing neural network (ProteinMPNN) was used to generate 64 unique sequences (maximum allowed), thereby increasing the likelihood of discovering a well-folded candidate. Sequence generation was initialized from the diffused backbone (initial_guess = True), which improves the sequence-to-structure compatibility. Cysteine residues were excluded (rm_aa = “C”) to avoid introducing disulfide-dependent stabilization into early binder designs. This was done to favor soluble designs whose folded state and target engagement would be less dependent on oxidation state, particularly given that the targeted SNAIL1 region was selected on the basis of redox-sensitive disorder behavior. Excluding cysteine also reduced the risk of undesired intramolecular or intermolecular disulfide formation that could complicate downstream interpretation of binder stability, folding, or specificity.

All generated sequences were subsequently evaluated using AlphaFold2 with 12 recycles for enhanced structure prediction confidence. The AlphaFold-multimer model was not used (use_multimer = False) because the system consisted of a single protein–binder pair. The best-performing model was selected based on the lowest root mean square deviation (RMSD) between the AlphaFold-predicted structure and the original RFdiffusion backbone, ensuring preservation of the intended fold. Here, RMSD refers to whole-binder structural deviation rather than an interface-restricted RMSD metric. While additional AlphaFold metrics (predicted local distance difference test [pLDDT], interface predicted alignment error, and interface predicted template modeling score) were also considered, RMSD was used as a comparative ranking metric across the generated design set rather than as a stand-alone indicator of experimental-quality structural convergence.

The complete set of 64 RFdiffusion-designed binder sequences is provided in File S1 (available at www.ophthalmologyscience.org), and all 64 RFdiffusion-generated binder structures are provided as a design ensemble in Dataset S1 (available at www.ophthalmologyscience.org). The AlphaFold-validated structure of the top-ranked binder design selected from this ensemble is provided in File S2 (available at www.ophthalmologyscience.org).

Our study was conducted using exclusively publicly available, deidentified data sources and computational modeling approaches, including protein sequence and structural data obtained from publicly accessible databases (e.g., UniProtKB, AlphaFold Protein Structure Database, and STRING). No human participants, patient-level data, or identifiable personal information were involved. As such, this work does not meet the definition of human subjects research and did not require institutional review board review. Accordingly, informed consent was not required. All procedures adhered to the tenets of the Declaration of Helsinki.

## Results

### Identification of a Target Protein

A total of 25 proteins were identified as being implicated in the pathogenesis of PVR, based on their roles in EMT, extracellular matrix remodeling, inflammation, or cell cycle regulation (Table 1). These included transcription factors (e.g., SNAIL1, Twist, MeCP2), signaling molecules (e.g., TGF-β, β-catenin, and METTL3), cytoskeletal and extracellular matrix components (e.g., alpha–smooth muscle actin, Vimentin, and FN1), and modulators of fibrosis and cell motility (e.g., YAP, RhoA/ROCK, TSPAN4, and epithelial membrane protein-2). The list also encompassed both profibrotic and antifibrotic regulators, such as ASPP2, DAPL1, and PPAR-γ, as well as microRNA targets (e.g., miR-4516 and OTX1). This curated panel of proteins served as the initial input for downstream computational analyses.

**Table 1:** Proteins Implicated in the Pathogenesis of PVR, along with Brief Descriptions of Their Roles

Protein	Function in PVR
TGF-β	Regulates EMT and fibrosis, initiating fibrotic pathways through Smad2/3 activation
α-SMA	Indicates mesenchymal state and promotes contractility of fibrotic membranes
Snail	Suppresses epithelial markers, promoting mesenchymal phenotype during EMT
Twist	Promotes mesenchymal markers, aiding EMT progression and fibrotic changes
MeCP2	Enhances TGF-β-induced EMT via Smad2/3 activation
β-Catenin	Facilitates EMT by promoting cell adhesion and migration
METTL3	Modulates Wnt/β-catenin, reducing EMT by downregulating Cyclin D1
Cyclin D1	Involved in cell cycle; downregulated by METTL3 in EMT inhibition
HGF	Induces EMT through receptor c-Met
c-Met	HGF receptor, facilitates EMT and cell migration
miR-4516	Targets OTX1, reducing EMT
OTX1	Promotes EMT when upregulated by TGF-β
RhoA/ROCK	Controls cytoskeletal reorganization, promoting cell motility and EMT
ASPP2	Inhibits TGF-β-induced EMT and autophagy
DAPL1	Stabilizes P21 to counter EMT
PPAR-γ	Antifibrotic regulator, downregulated in TGF-β-induced fibrosis
TSPAN4	Marker for migrasomes, promotes RPE cell migration and EMT
YAP	Promotes EMT by activating fibrosis markers under stress
EMP2	Regulates integrins, aiding cell adhesion and EMT signaling
COUP-TF1	Promotes EMT and cell migration; regulates transcriptional programs
E-cadherin	Cell adhesion molecule; loss promotes mesenchymal state
Vimentin	Supports structural cell integrity; increases during EMT
p53	Regulates cell cycle and apoptosis; controls RPE migration and adhesion
FN1 (Fibronectin 1)	Major ECM protein; promotes fibrosis
SPARC	Involved in ECM remodeling; upregulated in PVR membranes

ECM = extracellular matrix; EMT = epithelial–mesenchymal transition; EMP2 = epithelial membrane protein-2; PVR = proliferative vitreoretinopathy; RPE = retinal pigment epithelium; YAP = Yes-associated protein.

These proteins were selected based on their involvement in epithelial–mesenchymal transition (EMT), ECM remodeling, inflammation, or cell cycle regulation. They represent candidate molecules for mechanistic studies and therapeutic targeting in fibrosis-driven retinal diseases.

Canonical primary amino acid sequences for the identified EMT-related proteins were successfully retrieved from the UniProt. Using the UniProt ID mapping tool, we obtained unique UniProt accession numbers, ensuring selection of only *Homo sapiens* sequences. The FASTA sequences were formatted and stored for downstream computational analysis (Figure S1, available at www.ophthalmologyscience.org).

### Druggability Assessment

Our four-stage framework, intrinsic disorder evaluation, DOT quantification, biological relevance filtering, and STRING network assessment, established the objective criteria applied throughout the analysis. By defining explicit thresholds for percent disorder (30%–80%), redox-sensitive Δ disorder ≥0.20, and high-confidence PPI coherence, Table 2 serves as the operational foundation of the screening pipeline and ensures reproducibility of the filtering process. Table S1 details the full dataset of 25 EMT-associated proteins that underwent this stepwise evaluation. Each protein’s intrinsic disorder range, redox-dependent Δ disorder value, and RIDAO-derived metrics are reported, documenting which candidates were accepted or excluded.

**Table 2:** Quantitative Framework for Disorder-Based Druggability Assessment of EMT-Associated Proteins in PVR

Stage	Computational Tools and Metrics	Quantitative Parameters/Thresholds	Outcome and Rationale
1. Intrinsic Disorder Evaluation	RIDAO	% Disorder (30%–80% acceptable range); Excluded: <20% (structured) or >85% (fully disordered)	Identified proteins with balanced structural flexibility and potential for local ordering
2. DOT Region Identification	AIUPred (redox conditions)	Δ disorder ≥ 0.20 between redox-plus and redox-minus states	Quantified redox-sensitive disorder-to-order transitions, identifying flexible regions with conformational adaptability
3. Biological Relevance Filtering	Literature-supported EMT/PVR association	Retained proteins implicated in EMT, fibrosis, or PVR signaling pathways	Focused analysis on biologically relevant and disorder-eligible proteins
4. Network Assessment	STRING	Least stringent, limited interactome density	Validated biological coherence of prioritized proteins within PVR signaling networks

DOT = disorder-to-order transition; EMT = epithelial–mesenchymal transition; PVR = proliferative vitreoretinopathy; RIDAO = Rapid Intrinsic Disorder Analysis Online; STRING = Search Tool for the Retrieval of Interacting Genes/Proteins.

The analysis was conducted in four sequential stages: (1) intrinsic disorder evaluation using RIDAO to determine percent disorder and identify proteins with balanced structural flexibility; (2) DOT analysis using AIUPred under redox conditions to quantify conformational adaptability, including Δ disorder between redox-plus and redox-minus states; (3) biological relevance filtering to retain proteins supported by literature evidence for involvement in EMT, fibrosis, or PVR signaling; and (4) STRING v11.5 network assessment to confirm pathway coherence among prioritized candidates. Quantitative thresholds are shown for each stage to ensure reproducibility and standardization across the assessment.

Among the 25 EMT-associated candidate proteins evaluated for druggability, an initial screening using the RIDAO platform identified a broad distribution of intrinsic disorder percentages, ranging from highly ordered to completely disordered proteins. Proteins exhibiting very low disorder (<20%), such as Kallikrein, epithelial membrane protein-2, and TSPAN4, were excluded due to their rigid, globular nature, which limits conformational adaptability and interaction-driven folding. Conversely, proteins with extreme disorder (>85%), such as MeCP2, were removed from further analysis because of their lack of stable structural elements and low likelihood of forming defined binding interfaces. Although several EMT-associated proteins were retained at intermediate stages of screening, SNAIL1 emerged as the top-priority candidate because it satisfied the combined disorder, DOT, and network-based druggability criteria used in this study. Other biologically relevant EMT regulators, including YAP1 and TWIST, were considered during prioritization, but did not satisfy the same overall combination of screening criteria summarized in the supplementary tables. Accordingly, SNAIL1 was selected as the most coherent candidate for downstream structural targeting and binder design.

After applying the 30% to 80% intrinsic disorder inclusion threshold, the remaining proteins underwent further evaluation for DOT potential using AIUPred’s redox-sensitive prediction mode. Proteins displaying a redox-dependent disorder difference (Δ disorder) of less than 0.20 between redox-plus and redox-minus conditions were excluded due to insufficient conformational responsiveness. This filtering step removed several structurally stable extracellular matrix and cytoskeletal components, such as fibronectin and alpha–smooth muscle actin, which maintained high order and low redox sensitivity.

The refined subset included proteins with intermediate disorder and measurable DOT behavior, including SNAIL1, SPARC, YAP1, TWIST1, E2F1, Vimentin, PDPN, and E-cadherin. Among these, SNAIL1 exhibited the most pronounced Δ disorder and the strongest alignment between predicted DOT regions and known functional motifs, and the most promising STRING analysis.

**Figure 1:**
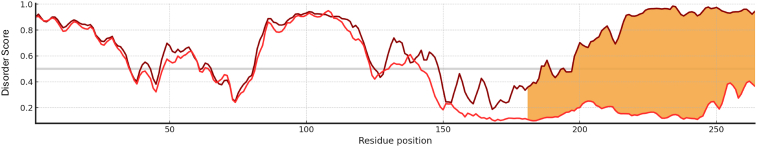
AIUPred-predicted redox-sensitive disorder-to-order transition (DOT) region in SNAIL1. AIUPred disorder propensity profiles for SNAIL1 under oxidizing (redox-plus; dark red trace) and reducing (redox-minus; bright red trace) conditions. The orange-shaded region highlights residues whose predicted disorder is reduced under oxidizing conditions, marking a redox-sensitive DOT segment. The horizontal 0.5 threshold line (gray) demarcates residues predicted to be intrinsically disordered.

SNAIL1 met or exceeded all predefined selection criteria (Table 3). The RIDAO classification identified 34.85% of SNAIL1 residues as intrinsically disordered, with predictions ranging from 28.03% (IUPred Long**)** to 65.91% (VSL2B). AIUPred redox-dependent analysis identified a prominent DOT region within the c-terminus of the protein, which exhibited a substantial reduction in disorder score under binding conditions (Fig 1; Δ disorder = 0.25; redox-plus: 0.64, redox-minus: 0.39).

**Table 3:** Quantitative and Qualitative Metrics Used to Evaluate the Druggability of SNAIL1 in the Context of Proliferative Vitreoretinopathy (PVR)

Category	Metric/Observation	Quantitative Value(s)
Intrinsic disorder content	PPDR from RIDAO meta-predictor (VLXT, VSL2B, VL3, IUPred-S/L, PFIT, MDP)	34.85% (MDP); range 28.03%–65.91%
Disorder-to-order transitions (DOTs)	DOT region overlapping with known DNA-binding residues.	Δ disorder = 0.25 (redox-plus: 0.64, redox-minus: 0.39)
Functional relevance in EMT	Literature-documented EMT regulator; represses E-cadherin, promotes ECM remodeling	NA - Qualitative
STRING PPI analysis	High-confidence (≥0.900) interactors; selective connectivity	21 interactors; GO terms: cadherin binding, TGF-β
Functional annotation	GO enrichment and clustering	EMT-related transcriptional and chromatin regulators

DOT = disorder-to-order transition; ECM = extracellular matrix; EMT = epithelial–mesenchymal transition; GO = gene ontology; MDP = mean disorder profile; PPDR = predicted percentage of disordered residue; PPI = protein–protein interaction; RIDAO = Rapid Intrinsic Disorder Analysis Online; STRING = Search Tool for the Retrieval of Interacting Genes/Proteins.

Metrics include intrinsic disorder content from the RIDAO meta-predictor, DOTs identified using AIUPred redox-dependent analysis, literature-based functional relevance to EMT, high-confidence PPI data from STRING, and functional annotation via GO enrichment.

**Figure 2:**
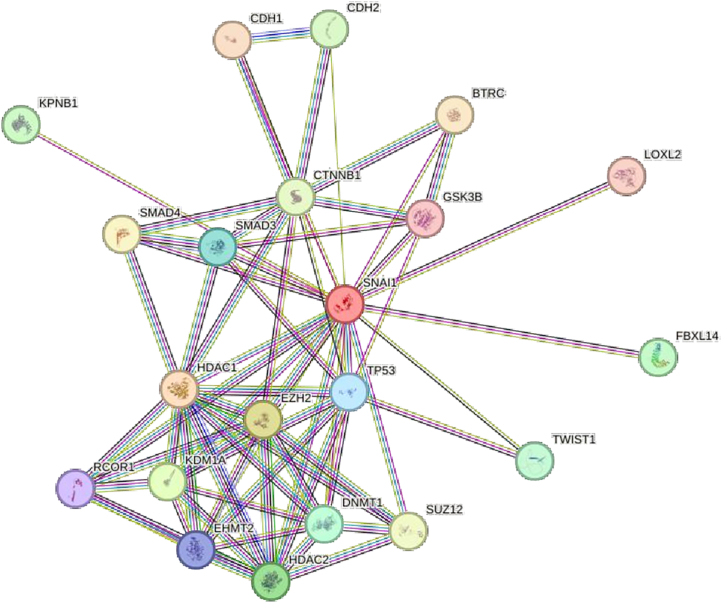
The STRING interaction network of SNAIL1. High-confidence (score ≥ 0.900) protein–protein interaction network generated in STRING v11.5. Node size reflects interaction degree; edge thickness reflects interaction confidence. Core EMT interactors (e.g., SMAD3/4, CTNNB1, and HDAC1/2) cluster around SNAIL1. STRING = Search Tool for the Retrieval of Interacting Genes/Proteins.

The STRING PPI analysis (minimum required interaction score ≥0.900) identified 21 high-confidence interactors, predominantly EMT-associated transcription factors and signaling regulators (Fig 2). Network enrichment analysis within STRING revealed overrepresentation of gene ontology terms related to cadherin binding, chromatin organization, and TGF-β signaling. Importantly, the network topology suggested selective connectivity rather than indiscriminate hub behavior, supporting its potential for specific therapeutic modulation.

### Evaluation of SNAIL1

**Figure 3:**
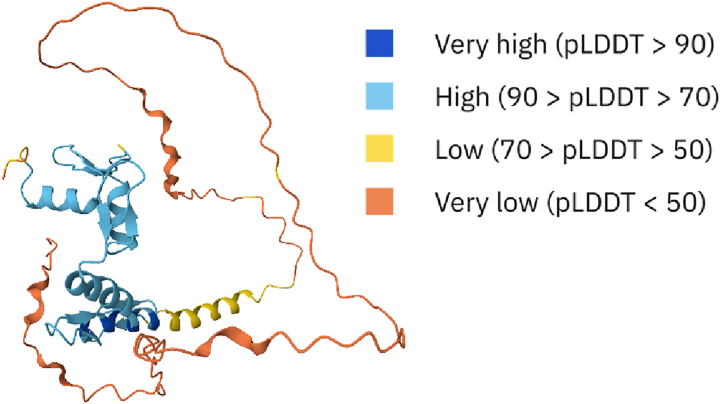
Predicted structure of SNAIL1 generated by AlphaFold. The structure is color-coded by per-residue confidence according to the pLDDT: blue indicates high confidence (>90), yellow indicates moderate confidence (70–90), and orange to red indicates low confidence (<70). A well-defined α-helical domain with high pLDDT values is visible near the center of the structure, while extended coil regions at the N-termini and C-termini are associated with lower prediction confidence. pLDDT = predicted local distance difference test.

The AlphaFold-predicted structure of SNAIL1 revealed a distinct modular organization characterized by a compact, well-ordered core flanked by extended, flexible termini (Fig 3). The central region formed a β-sheet and α-helical elements characteristic of C2H2 zinc fingers corresponding to the zinc-finger domain, rendered in blue to denote high pLDDT confidence (>90). This domain occupies the geometric center of the protein, forming a relatively globular structural nucleus that likely stabilizes the overall fold and anchors interdomain interactions. Its well-defined α-helical packing suggests a stable scaffold for coordinating zinc ions and maintaining spatial orientation for DNA or cofactor binding. In contrast, the N- and C-terminal extensions appeared as elongated coil-like segments with lower pLDDT values (yellow to orange), consistent with intrinsic disorder and conformational plasticity. These flexible termini may confer dynamic adaptability, enabling SNAIL1 to engage multiple binding partners and undergo redox-sensitive DOTs that modulate its activity.

Literature-guided annotation of the AIUPred predicted DOT region (residues 181–264) highlighted a cluster of basic residues with convergent evidence for regulatory and interaction-related roles (Table 4). Prior structural and functional studies have established that the C-terminal domain of SNAIL1, spanning approximately residues 151 to 264, contains a series of C2H2-type zinc-finger motifs that mediate DNA binding and simultaneously function as a nuclear localization signal through conserved basic residues recognized by importin family members.^46^^,^^47^ In this context, we utilized peer-reviewed studies to identify potential regulatory sites within the 181 to 264 DOT window.

**Table 4:** Functional Mapping of Key Basic Residues within the SNAIL1 DOT Region

Residue	Experimental/Theoretical Evidence	Key Supporting Findings	Representative References	Functional Interpretation
K187	Experimentally validated PTM site	Acetylated by CBP/p300, which increases stability and transcriptional activity by preventing ubiquitination. Located near N-terminal edge of DOT region, anchoring a regulatory interface.	Dong & Wu, 2021; Peinado et al, 2019; Wu et al, 2017	N-terminal “anchor” residue; defines one edge of the DOT epitope and marks a known regulatory PTM site.
R191	Theoretical (motif-based)	Positively charged residue adjacent to K187 within basic stretch implicated in DNA binding and nuclear localization; conserved among SNAIL1 family orthologs.	Huret et al, 2013; Choi et al, 2014	Extends electropositive patch from K187; likely contributes to importin-β or DNA interaction surface.
R224	Theoretical (structural/homology)	Lies in middle of DOT region; aligns with DNA-contact residues in C2H2-type zinc-finger repressors; contributes to continuous electropositive surface.	Huret et al, 2013; Dong & Wu, 2021	Central basic contact bridging N- and C-terminal subsegments of the DOT region.
K234	Experimentally validated PTM site	SUMOylated and ubiquitinated under TGF-β signaling; modifications promote nuclear retention and stability of SNAIL1 during EMT.	Dong & Wu, 2021; Gudey et al, 2017; Chang et al, 2018	Distal regulatory “anchor”; central hub linking extracellular signaling to SNAIL1 nuclear function.
K253	Theoretical (motif/structural)	Basic residue near distal zinc-finger helix; part of C-terminal nuclear localization signals (NLS)/DNA-binding motif in Snail family proteins.	Huret et al, 2013; Choi et al, 2014	Extends basic interface toward C-terminus; maintains continuity of the electropositive surface.
R264	Theoretical (motif/functional homology)	Terminal residue of SNAIL1; component of basic tail required for importin-β–dependent nuclear import and DNA interaction.	Huret et al, 2013; Dong & Wu, 2021	Defines the terminal boundary of the DOT epitope; ensures full coverage of the C-terminal nuclear-localization surface.

DOT = disorder-to-order transition; EMT = epithelial–mesenchymal transition; PTM = post-translational modification.

Our review identified Lys187 as a recurrently reported acetylation site targeted by CREB-binding protein (CBP)/p300. Multiple independent studies using co-immunoprecipitation and mass spectrometry have demonstrated that K187 (often in conjunction with K146) is acetylated by CBP and p300, thereby enhancing SNAIL1 transcriptional activity, reducing its ubiquitination, and stabilizing the protein in cancer cells.^48^^,^^49^ Given that K187 lies within the C-terminal DOT region and is positively charged, these findings support its designation as a regulatory “anchor” residue at the N-terminal edge of the DOT window.

A second strongly supported position within the DOT region, Lys234, has been characterized as a SUMOylation site whose modification is induced by TGF-β signaling. Sumoylation of K234 promotes SNAIL1 nuclear accumulation, stability, and pro-invasive function, and perturbation of this site attenuates EMT-related phenotypes in multiple carcinoma models.^48^^,^^50^ These data identify K234 as a regulatory hub within the C-terminal domain, linking extracellular TGF-β signals to SNAIL1 nuclear function.

In contrast, Arg191, Arg224, Lys253, and Arg264 have not been as extensively studied individually at the PTM level. However, they occur within or adjacent to motifs that have been implicated in SNAIL1 DNA binding and nuclear localization. Work on SNAIL1 family proteins and related C2H2 transcription factors has demonstrated that basic residues positioned at defined sites across consecutive zinc fingers and within the distal C-terminal tail form a conserved nuclear localization signal and contribute to specific DNA recognition by the zinc-finger array.^47^ These residues are positively charged, lie within the same C-terminal functional block, and align with consensus patterns described for importin-β–dependent nuclear import and E-box binding.

K187 and K234 are directly validated regulatory lysines with well-defined acetylation and SUMOylation roles, whereas R191, R224, K253, and R264 extend this regulatory core along the basic C-terminal surface implicated in DNA binding and nuclear localization. By restricting candidate sites to residues with either (1) experimentally demonstrated PTM/functional roles or (2) strong theoretical support from conserved C-terminal motifs in the SNAIL1 family, this literature-guided mapping provided a focused, mechanistically grounded set of hot-spot candidates. These 6 residues were therefore selected as the design epitope for RFdiffusion-based binder generation (section RFdiffusion-Based Binder Design), with the working hypothesis that engaging this PTM and localization-enriched basic surface could modulate SNAIL1 activity without disrupting the structural zinc-coordination core.

### RFdiffusion-Based Binder Design for SNAIL1

**Figure 4:**
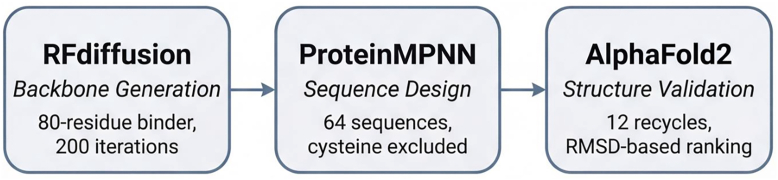
Schematic overview of the computational binder-design workflow. Following disorder, DOT, and network-based prioritization of EMT-associated proteins, SNAIL1 was selected as the top target. Six candidate interface residues within the SNAIL1 DOT region (K187, R191, R224, K234, K253, and R264) were used to guide RFdiffusion-based de novo binder backbone generation. The resulting backbones were sequence-optimized with ProteinMPNN and structurally evaluated using AlphaFold2. Candidate binders were then ranked using structural and interface-related metrics, including RMSD, pLDDT, iPAE, and iPTM. EMT = epithelial–mesenchymal transition; DOT = disorder-to-order transition; iPAE = interface predicted alignment error; iPTM = interface predicted template modeling score; pLDDT = predicted local distance difference test; ProteinMPNN = protein message passing neural network; RMSD = root mean square deviation.

**Figure 5:**
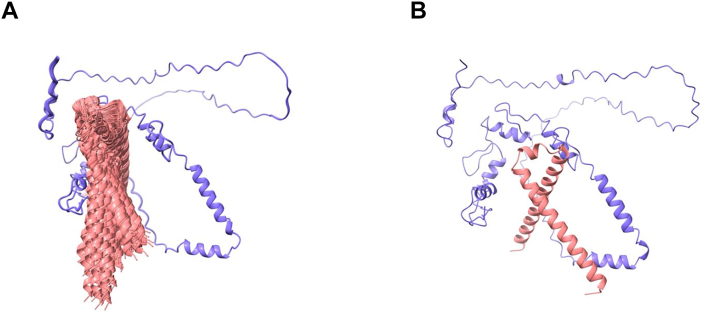
Structures generated with RFDiffusion pipeline. **A,** Overlay of 64 RFdiffusion-generated binder backbones (red) onto the SNAIL1 target (blue), showing the ensemble of structural hypotheses before sequence design. **B,** Overlay of the top-ranked binder structure, after ProteinMPNN sequence design and AlphaFold2 validation, docked onto SNAIL1 (blue). ProteinMPNN = protein message passing neural network.

As outlined in Figure 4, the selected SNAIL1 interface residues were used in a sequential design pipeline consisting of RFdiffusion backbone generation, ProteinMPNN sequence assignment, and AlphaFold2-based model evaluation. As shown in Figure 5, RFdiffusion generated an ensemble of 64 backbone conformations (red) overlayed on SNAIL1 (blue) (Fig 5A), illustrating the conformational diversity of our initial designs. Each panel visualizes the predicted binding pose: panel A captures this variation across the design space, while panel B shows the single, highest confidence complex retained after ProteinMPNN sequence design and AlphaFold2 validation (Fig 5B). The validated binder maintained its overall fold and continued to engage the DOT region, though its position was slightly shifted relative to the initial design ensemble. This minor deviation likely reflects the influence of sequence-dependent effects introduced during AlphaFold2 refinement. Importantly, the persistence of binding within the DOT region supports the ability of our epitope-guided design pipeline, biased toward AIUPred-identified residues, to produce stable, structure-aware candidates with region-specific targeting.

**Figure 6:**
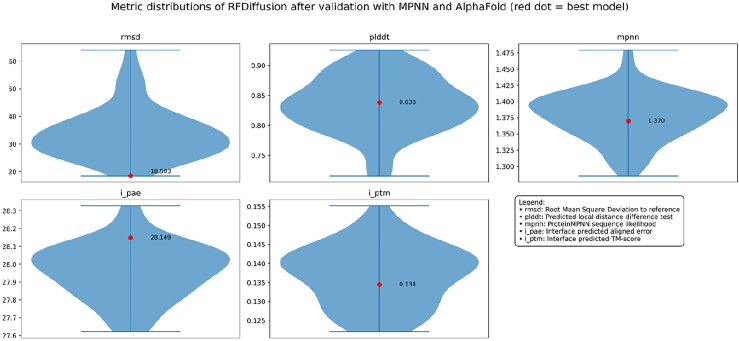
Violin plots of design quality metrics for RFdiffusion outputs. Red dots indicate the selected best model as determined by the lowest RMSD, validated using MPNN sequence likelihood and AlphaFold structure predictions. MPNN = message passing neural network; RMSD = root mean square deviation.

Among all generated designs, model n = 33 exhibited the lowest RMSD (18.503) relative to the RFdiffusion-designed backbone and was selected as the top candidate. The violin plots in Figure 6 summarize the distribution of key design-quality metrics across all 64 RFdiffusion-generated models following ProteinMPNN sequence optimization and AlphaFold2 structural validation. These metrics collectively quantify structural fidelity, sequence compatibility, and interface precision, offering a multidimensional view of model performance across the entire design ensemble. Among them, RMSD was used primarily as a relative ranking metric to identify the design that deviated least from its original RFdiffusion backbone after AlphaFold2 refinement. Although the absolute RMSD remained high, model 33 represented the most structurally self-consistent candidate within the generated set rather than a near-native or experimentally validated binder conformation. Its mean pLDDT score (0.838) indicated relatively strong per-residue confidence in the predicted binder fold, while the ProteinMPNN sequence-likelihood score (1.370) supported compatibility between the designed sequence and scaffold. Interface-related metrics, including interface predicted alignment error (28.149) and interface predicted template modeling score (0.134), were retained as supplementary computational descriptors of predicted complex organization rather than as stand-alone indicators of binder quality. No standardized reference set of experimentally validated de novo binders targeting intrinsically disordered regions currently exists to enable direct numerical benchmarking of these values. However, a recent large-scale meta-analysis of 3766 experimentally characterized de novo binders demonstrated that, while interface-focused metrics are informative for binder prioritization, interface predicted alignment error and interface predicted template modeling score are generally outperformed by more interface-restricted confidence measures in predicting experimental success,^51^ supporting a cautious interpretation of these scores in the present context.

## Discussion

Our study establishes a novel disorder-informed computational framework for identifying and characterizing EMT proteins implicated in PVR, offering the first systematic assessment of structural plasticity as a determinant of therapeutic tractability in this disease. By integrating multitool disorder prediction (RIDAO and AIUPred), functional pathway filtering, STRING network assessment, and AI-driven binder design (RFdiffusion + AlphaFold2), we demonstrate that SNAIL1, an EMT transcription factor, is uniquely positioned as a druggable, intrinsically disordered regulator of fibrotic remodeling in PVR. The analysis revealed that SNAIL1 exhibits moderate intrinsic disorder (∼35%), a distinct redox-sensitive disorder-to-order SNAIL1 (Δ disorder = 0.25) localized to its C-terminal zinc-finger domain, and selective network connectivity within EMT-related pathways, fulfilling all 4 pillars of our quantitative druggability assessment. These features, together with a mechanistically anchored and structure-guided set of candidate interface residues (K187, R191, R224, K234, K253, and R264) within the DOT region, enabled the generation of a structure-aware RFdiffusion-designed binder, validating the potential of AI-guided disorder targeting as a frontier for antifibrotic drug discovery in PVR. Broadly, this study introduces an adaptable pipeline that redefines PVR therapeutic development, shifting from empirical inhibitor screening toward rational, disorder-based design guided by deep learning structural predictions.

Functionally, SNAIL1 is a master regulator of EMT, directly repressing E-cadherin and promoting extracellular matrix remodeling, both central to PVR pathogenesis^18^^,^^25^ Its activation downstream of TGF-β/Smad signaling and cooperation with Wnt/β-catenin and ERK1/2–mitogen-activated protein kinase cascades drive the transformation of RPE cells into contractile myofibroblast-like cells, hallmarks of fibrotic membrane formation.^19^^,^^20^^,^^26^^,^^52^

Our disorder-based structural analysis adds mechanistic context to SNAIL1’s role in PVR by identifying that the C-terminal DOT region (residues 181–264) overlaps regulatory motifs modified by acetylation at K187 (by CREB-binding protein/p300) and SUMOylation at K234 (under TGF-β stimulation), PTMs that stabilize SNAIL1 and enhance transcriptional activity.^48^^,^^49^^,^^53^ Importantly, acetylation at K187 has been previously shown to enhance SNAIL1’s transcriptional potency by promoting nuclear retention and stabilizing its interaction with co-activators such as CBP/p300, highlighting this residue as a functional hot spot within the DOT interface.^54^^,^^55^ Targeting this interface could theoretically disrupt CBP/p300-mediated stabilization, promoting SNAIL degradation and restoring epithelial polarity. This mechanistic linkage is consistent with prior observations that CBP-driven acetylation enhances SNAIL1’s DNA-binding affinity and prolongs its half-life.^49^ Thus, inclusion of K187 as an anchor residue in our binder design provides a rational, literature-supported target to disrupt CBP/p300 binding and modulate SNAIL1 turnover. Collectively, these results suggest that our binder design, focused on this PTM-enriched DOT surface, may interfere with SNAIL1’s CBP/p300 interaction, representing a rational structural entry point for modulating EMT in fibrotic retinal disease.

Although K187 and K234 represent experimentally validated PTM sites with defined regulatory consequences, the remaining residues incorporated into the binder interface (R191, R224, K253, and R264) lack direct residue-specific functional validation. Their inclusion was guided instead by conserved motif architecture and structural context, as these basic residues localize within the C-terminal zinc, where clusters of positively charged residues are known to mediate nuclear import, DNA binding, and co-factor engagement. AlphaFold-derived structural models were used cautiously as hypothesis-generating tools to identify spatial clustering and surface accessibility within this DOT region, recognizing that such predictions do not capture PTMs, conformational ensembles, or dynamic disorder behavior. Together, this distinction between experimentally validated anchor sites and theoretically supported supporting contacts preserves mechanistic rigor while providing a coherent, structure-informed interface for exploratory binder design.

Despite EMT’s centrality, PVR pathogenesis involves a broader pathological framework that includes chronic inflammation, immune-cell infiltration, glial activation, and extracellular matrix remodeling, as well as other transition programs such as endothelial–mesenchymal transition and macrophage–myofibroblast transition. Persistent cytokine signaling through TGF-β, platelet-derived growth factor, VEGF, connective tissue growth factor, and tumor necrosis factor alpha amplifies these fibrotic loops and sustains cellular activation.^56^^,^^57^ Within this integrated model, EMT represents the best-characterized and experimentally validated driver of fibrotic remodeling, serving as the nexus that links inflammatory cues to extracellular matrix overproduction and contraction. By positioning SNAIL1 within this multipathway network, our findings reinforce its role as a central transcriptional integrator, bridging these cellular processes. Modulating its conformational flexibility could, in principle, attenuate downstream EMT, endothelial–mesenchymal transition, and macrophage–myofibroblast transition cascades, yielding broad antifibrotic potential. In a translational context, such a binder-based therapeutic could be envisioned as an adjunctive perioperative strategy, administered intraoperatively at the time of RD repair or postoperatively during the critical wound-healing phase, to suppress EMT-driven membrane recurrence and improve long-term anatomic outcomes.

Transcription factors such as SNAIL1 have long been considered “undruggable,” largely because they lack deep hydrophobic pockets typical of enzyme targets. However, recent studies underscore that intrinsically disordered regions frequently harbor short linear motifs that mediate transient yet essential regulatory contacts^58^, ^59^, ^60^ (Holehouse & Kragelund 2024 [29]; Uversky 2024 [28]). Our framework makes use of this emerging principle by integrating redox-sensitive disorder prediction (AIUPred) with deep learning–based backbone generation and sequence design (RFdiffusion + ProteinMPNN + AlphaFold2), to pinpoint discrete amino acids within DOT subregions that function as transient, yet druggable, conformational interfaces, and to demonstrate that these specific residues can be structurally targeted using AI-guided binder design.

This approach is conceptually aligned with the recent work of Wu et al, which demonstrated that de novo-designed proteins can successfully bind dynamic intrinsically disordered region segments once thought intractable.^30^ By adapting this frontier to an ophthalmic disease context, our study provides an early demonstration that deep learning models can uncover latent druggability in fibrotic regulators that were previously beyond reach.

Future experimental studies should evaluate both the biological relevance of the prioritized SNAIL1 interface and the activity of the designed binders in retinal systems relevant to PVR. In vitro validation could include RPE EMT assays to determine whether perturbation of the prioritized SNAIL1 DOT region alters epithelial and mesenchymal marker expression, cellular migration, contractility, or fibrotic behavior under EMT-inducing conditions such as TGF-β stimulation.^61^ A particularly informative next step would be residue-specific validation of K187 in RPE EMT systems. Because K187 is a literature-supported acetylation site linked to SNAIL1 stability and transcriptional activity, mutagenesis of this residue, for example, to a poorly acetylated substitute such as K187R, could help determine whether perturbation of this PTM-sensitive site alters EMT-associated phenotypes. Direct binding studies, including surface plasmon resonance, nuclear magnetic resonance, or related biophysical assays, would also be important to test whether the designed binder can engage the targeted SNAIL1 region with measurable affinity and specificity.^62^ Ultimately, in vivo evaluation in established experimental models of PVR will be required to determine whether modulation of this interface can reduce fibrotic membrane formation or improve anatomic outcomes in a disease-relevant setting.

Beyond its mechanistic implications, this framework raises important considerations for the clinical management of PVR. From a clinical perspective, the ability to modulate EMT upstream of membrane formation has important implications for preventing PVR. Current adjunctive strategies for high-risk RD act broadly and have yielded inconsistent efficacy. Targeting EMT regulators, such as SNAIL1, offers a mechanistically distinct approach to interrupt the transcriptional programs that drive RPE differentiation and fibrotic membrane formation. While the present study is entirely computational, the identification of structurally tractable DOT regions raises the possibility of developing perioperative or early postoperative interventions aimed at patients at highest risk for PVR, such as those with large or chronic detachments, trauma, or prior surgical failure.

### Limitations

Despite the strengths of our integrative computational approach, several limitations should be acknowledged to contextualize our findings. While this study focuses on EMT as the primary mechanism underlying PVR, it is important to acknowledge that alternative pathogenic mechanisms have been proposed. Other theories, such as inflammation-driven fibrosis, metabolic dysregulation, and oxidative stress**,** may also play significant roles in PVR progression. By centering our analysis on EMT-related proteins, we may have overlooked key contributors involved in non-EMT-driven fibrotic pathways.

A related conceptual limitation is that the amino acid residues prioritized in our binder design, particularly K187 and R264, have been experimentally validated in cancer or EMT models, but not directly in the context of PVR. While this may appear to limit direct disease-specific relevance, the overlap in underlying biology is strong: EMT represents a unifying pathological program between neoplasia and fibrosis. Thus, functional inferences drawn from oncologic models likely translate to PVR, where EMT drives RPE transformation, matrix contraction, and membrane formation. This extrapolation, though biologically justified, nonetheless underscores the need for empirical validation of residue-specific functions within ocular tissue systems.

Relatedly, the 6 candidate interface residues were prioritized using a static AlphaFold-predicted structure, without explicit characterization of their dynamic solvent accessibility or conformational variability across the intrinsically disordered ensemble. Because IDPs sample a heterogeneous conformational landscape, residues that appear surface-exposed in a single predicted structure may be transiently buried or conformationally restricted in the native ensemble, and vice versa. While the successful generation of RFdiffusion binder backbones engaging these residues, with subsequent AlphaFold2 confirmation of the predicted complex, supports their structural plausibility as an interface in the modeled conformation, this does not substitute for ensemble-level validation. Future work should incorporate molecular dynamics simulations or enhanced sampling methods to quantify per-residue solvent-accessible surface area distributions, side-chain rotameric flexibility, and transient pocket formation within the DOT region across the conformational ensemble. Such analyses would enable a more rigorous assessment of which candidate residues are consistently accessible and structurally favorable for binder engagement, allowing refinement of the epitope definition prior to subsequent rounds of experimental or computational design.

Future experimental studies will be required to validate the functional relevance of individual interface residues and to refine or narrow the epitope definition based on empirical binding and phenotypic data. Although our framework permits rapid reparameterization of these target positions, future iterations may benefit from refining or narrowing the epitope set to experimentally supported residues. In addition, serine and threonine residues, specifically T203, S246, and S249, represent untested but potentially relevant PTM sites that warrant future exploration. Given that phosphorylation at these residues could modulate SNAIL1 conformation and activity, incorporating them into the next design iteration could enhance the biological realism of the targeting approach.

Additionally, this research is entirely computational, relying on previously published datasets and in silico predictions rather than direct experimental validation. Our findings are based on structural modeling, RFDiffusion simulations, and disorder-based analyses, all of which are dependent on the accuracy of available protein structures and the predictive power of force fields and AI-driven tools. While we utilized state-of-the-art modeling platforms such as AlphaFold and RFDiffusion, predictions are inherently limited by training data and algorithmic assumptions**,** which may introduce biases or inaccuracies. In addition, the top-ranked binder displayed substantial structural deviation from the original RFdiffusion backbone (RMSD 18.5 Å), indicating that the current design should be interpreted as a preliminary computational lead rather than a structurally converged binder candidate. Similarly, the 80-residue binder length used in the RFdiffusion design was selected as a single representative scaffold size that balances structural stability with minimal conformational complexity, consistent with the general scale of experimentally tractable mini-protein binders; however, we acknowledge that this represents only 1 point within the feasible design space, and future work could evaluate alternative binder lengths or architectures to further optimize structural fit and binding affinity.

A further limitation is that, among the EMT-associated proteins retained at intermediate screening stages, only SNAIL1 was advanced to full structural characterization and binder design. Other biologically relevant candidates, including YAP1 and TWIST1, were deprioritized because they did not satisfy the same combination of disorder, DOT magnitude, and network-based druggability criteria applied in our staged workflow. However, this does not exclude the possibility that these proteins harbor targetable disordered interfaces through mechanisms not captured by our current screening parameters. Comparative structural and druggability profiling of these candidates, including disorder-to-order mapping and interface residue identification, represents a valuable direction for future work and could reveal additional or alternative therapeutic entry points for PVR.

Another key limitation is the lack of experimental confirmation for our structural and druggability assessments. We have not yet performed wet-lab experiments, such as direct binding or functional assays in RPE systems, to validate our computational predictions. Future studies should therefore test whether perturbation of the prioritized SNAIL1 DOT/interface region alters EMT-associated phenotypes in vitro, whether the designed binder can directly engage this region with measurable affinity and specificity, and whether such modulation has therapeutic relevance in vivo in experimental PVR models. Nonetheless, this study establishes a structured computational framework that enables hypothesis-driven target identification and prioritization. While we acknowledge the absence of experimental insights, our in silico approach provides a rational starting point that can be reasonably tested and iterated upon. In fact, given the often-cited 1 in 20 000 success rate in traditional drug discovery pipelines, even low-probability computational leads such as those identified here warrant investigation. The goal is not to present definitive therapeutic targets, but to spark experimental validation and refinement by our group or others. Future studies should incorporate biophysical and biochemical experiments to confirm the structural relevance and therapeutic potential of the identified EMT-related proteins in PVR. Despite these limitations, our study provides a valuable computational framework for prioritizing potential drug targets and guiding experimental validation in future research.

## Conclusion

Proliferative vitreoretinopathy remains one of the most challenging complications in vitreoretinal surgery, with no effective pharmacologic therapy to prevent fibrotic recurrence or improve surgical outcomes. This study introduces a disorder-informed computational framework that reframes the search for PVR therapeutics through the lens of protein structural plasticity and AI-guided molecular design. By integrating intrinsic disorder prediction, redox-sensitive modeling, and generative binder design, we identify SNAIL1 as a tractable, intrinsically disordered master regulator of EMT, a central driver of retinal fibrosis. Importantly, this framework provides a rational foundation for future experimental interrogation of SNAIL1 modulation in established in vitro RPE EMT systems and in vivo PVR models, enabling direct evaluation of whether disorder-targeted interventions can attenuate fibrotic membrane formation. This work defines a new molecular rationale for targeting EMT in PVR and establishes a scalable, structure-aware discovery paradigm that can extend to other fibrotic eye diseases.

## Use of Generative AI and AI-Assisted Technologies in the Writing Process

During the preparation of this manuscript, the authors used generative artificial intelligence tools, including OpenAI’s ChatGPT and Google’s Gemini, to assist with language refinement and clarity of expression. All content generated with the assistance of these tools was critically reviewed, edited, and validated by the authors, who take full responsibility for the accuracy, integrity, and originality of the work.
