# Supplementary material for: A Disorder-Aware Computational Framework to Identify Structurally Tractable Targets in Proliferative Vitreoretinopathy
Source: Ophthalmol Sci. 2026 May 25;6(8):101249. doi: 10.1016/j.xops.2026.101249 (PMC13343151; doi:10.1016/j.xops.2026.101249)
Supplement: Table S1 [file mmc3.docx]

**Supplementary Table S1.** The 25 EMT-associated proteins were analyzed via RIDAO (intrinsic disorder and DOT parameters) and AIUPred (redox-sensitive disorder modeling).

| **Protein** | **% Intrinsic Disorder (RIDAO Range)** | **Δ Disorder (Redox⁺ − Redox⁻)** | **Outcome** | **Rationale** |
| --- | --- | --- | --- | --- |
| Kallikrein | 12–19% | <0.10 | Excluded | Highly structured serine protease (<20% disorder). No DOT. |
| EMP2 | 1–8% | <0.10 | Excluded | Transmembrane; minimal disorder (<20%). |
| TSPAN4 | 15–20% | <0.10 | Excluded | Rigid tetraspanin; lacks conformational adaptability. |
| Cyclin D1 | 16–25% | <0.10 | Excluded | Structured; no extended IDRs or DOT regions. |
| COUP-TF1 | 24–39% | <0.10 | Excluded | Moderately structured transcription factor; no DOT regions. |
| METTL3 | 32–47% | <0.10 | Excluded | Moderately structured enzyme; no redox sensitivity. |
| HGF1 | 1–31% | <0.10 | Excluded | Structured growth factor; lacks DOT regions. |
| Fibronectin (FN1) | 41–46% | 0.08 | Excluded | ECM protein; low Δ disorder (<0.20). |
| α-SMA (ACTA2) | 36–40% | 0.11 | Excluded | Cytoskeletal; limited conformational response. |
| ASPP2 | 49–79% | <0.10 | Excluded | Highly disordered; no DOT despite redox testing. |
| MeCP2 | 77–95% | 0.14 | Excluded | Extremely disordered (>85%); unstable for binding. |
| OTX1 | 52–81% | 0.18 | Excluded | Highly disordered transcription factor; Δ disorder <0.20. |
| CTGF | 48–52% | 0.17 | Excluded | Moderately disordered but fails redox responsiveness cutoff. |
| TGF-β | 42–55% | 0.19 | Excluded | Near cutoff; modest Δ disorder, insufficient DOT. |
| FN-EDA | 45–52% | 0.18 | Excluded | ECM variant; fails redox threshold. |
| SPARC | 32–47% | 0.22 | Retained | Balanced disorder (30–80%) and Δ disorder >0.20. |
| YAP1 | 70% | 0.24 | Retained | High disorder; strong DOT and EMT relevance. |
| TWIST1 | 69% | 0.21 | Retained | Significant DOT and transcriptional adaptability. |
| Vimentin | 74% | 0.20 | Retained | Balanced high disorder and structural adaptability. |
| E2F1 | 55–73% | 0.23 | Retained | Strong intrinsic disorder; high DOT response. |
| PDPN | 56–73% | 0.22 | Retained | Strong DOT; disordered extracellular region. |
| E-cadherin | 28–30% | 0.20 | Retained | Moderate disorder; dynamic adhesion and DOT potential. |
| SNAIL1 | 34–66% | 0.25 | Retained | Optimal disorder range and Δ disorder; top candidate. |
