# Supplementary material for: A Disorder-Aware Computational Framework to Identify Structurally Tractable Targets in Proliferative Vitreoretinopathy
Source: Ophthalmol Sci. 2026 May 25;6(8):101249. doi: 10.1016/j.xops.2026.101249 (PMC13343151; doi:10.1016/j.xops.2026.101249)
Supplement: Figure S1 [file mmc4.docx]

**Supplementary Figure S1**. FASTA-formatted amino acid sequences of PVR-associated proteins used for computational druggability analyses.

>sp|O95863|SNAI1_HUMAN Zinc finger protein SNAI1 OS=Homo sapiens OX=9606 GN=SNAI1 PE=1 SV=2

MPRSFLVRKPSDPNRKPNYSELQDSNPEFTFQQPYDQAHLLAAIPPPEILNPTASLPMLIWDSVLAPQAQPIAWASLRLQESPRVAELTSLSDEDSGKGSQPPSPPSPAPSSFSSTSVSSLEAEAYAAFPGLGQVPKQLAQLSEAKDLQARKAFNCKYCNKEYLSLGALKMHIRSHTLPCVCGTCGKAFSRPWLLQGHVRTHTGEKPFSCPHCSRAFADRSNLRAHLQTHSDVKKYQCQACARTFSRMSLLHKHQESGCSGCPR

>sp|P01137|TGFB1_HUMAN Transforming growth factor beta-1 proprotein OS=Homo sapiens OX=9606 GN=TGFB1 PE=1 SV=3

MPPSGLRLLPLLLPLLWLLVLTPGRPAAGLSTCKTIDMELVKRKRIEAIRGQILSKLRLASPPSQGEVPPGPLPEAVLALYNSTRDRVAGESAEPEPEPEADYYAKEVTRVLMVETHNEIYDKFKQSTHSIYMFFNTSELREAVPEPVLLSRAELRLLRLKLKVEQHVELYQKYSNNSWRYLSNRLLAPSDSPEWLSFDVTGVVRQWLSRGGEIEGFRLSAHCSCDSRDNTLQVDINGFTTGRRGDLATIHGMNRPFLLLMATPLERAQHLQSSRHRRALDTNYCFSSTEKNCCVRQLYIDFRKDLGWKWIHEPKGYHANFCLGPCPYIWSLDTQYSKVLALYNQHNPGASAAPCCVPQALEPLPIVYYVGRKPKVEQLSNMIVRSCKCS

>sp|P08581|MET_HUMAN Hepatocyte growth factor receptor OS=Homo sapiens OX=9606 GN=MET PE=1 SV=4

MKAPAVLAPGILVLLFTLVQRSNGECKEALAKSEMNVNMKYQLPNFTAETPIQNVILHEHHIFLGATNYIYVLNEEDLQKVAEYKTGPVLEHPDCFPCQDCSSKANLSGGVWKDNINMALVVDTYYDDQLISCGSVNRGTCQRHVFPHNHTADIQSEVHCIFSPQIEEPSQCPDCVVSALGAKVLSSVKDRFINFFVGNTINSSYFPDHPLHSISVRRLKETKDGFMFLTDQSYIDVLPEFRDSYPIKYVHAFESNNFIYFLTVQRETLDAQTFHTRIIRFCSINSGLHSYMEMPLECILTEKRKKRSTKKEVFNILQAAYVSKPGAQLARQIGASLNDDILFGVFAQSKPDSAEPMDRSAMCAFPIKYVNDFFNKIVNKNNVRCLQHFYGPNHEHCFNRTLLRNSSGCEARRDEYRTEFTTALQRVDLFMGQFSEVLLTSISTFIKGDLTIANLGTSEGRFMQVVVSRSGPSTPHVNFLLDSHPVSPEVIVEHTLNQNGYTLVITGKKITKIPLNGLGCRHFQSCSQCLSAPPFVQCGWCHDKCVRSEECLSGTWTQQICLPAIYKVFPNSAPLEGGTRLTICGWDFGFRRNNKFDLKKTRVLLGNESCTLTLSESTMNTLKCTVGPAMNKHFNMSIIISNGHGTTQYSTFSYVDPVITSISPKYGPMAGGTLLTLTGNYLNSGNSRHISIGGKTCTLKSVSNSILECYTPAQTISTEFAVKLKIDLANRETSIFSYREDPIVYEIHPTKSFISGGSTITGVGKNLNSVSVPRMVINVHEAGRNFTVACQHRSNSEIICCTTPSLQQLNLQLPLKTKAFFMLDGILSKYFDLIYVHNPVFKPFEKPVMISMGNENVLEIKGNDIDPEAVKGEVLKVGNKSCENIHLHSEAVLCTVPNDLLKLNSELNIEWKQAISSTVLGKVIVQPDQNFTGLIAGVVSISTALLLLLGFFLWLKKRKQIKDLGSELVRYDARVHTPHLDRLVSARSVSPTTEMVSNESVDYRATFPEDQFPNSSQNGSCRQVQYPLTDMSPILTSGDSDISSPLLQNTVHIDLSALNPELVQAVQHVVIGPSSLIVHFNEVIGRGHFGCVYHGTLLDNDGKKIHCAVKSLNRITDIGEVSQFLTEGIIMKDFSHPNVLSLLGICLRSEGSPLVVLPYMKHGDLRNFIRNETHNPTVKDLIGFGLQVAKGMKYLASKKFVHRDLAARNCMLDEKFTVKVADFGLARDMYDKEYYSVHNKTGAKLPVKWMALESLQTQKFTTKSDVWSFGVLLWELMTRGAPPYPDVNTFDITVYLLQGRRLLQPEYCPDPLYEVMLKCWHPKAEMRPSFSELVSRISAIFSTFIGEHYVHVNATYVNVKCVAPYPSLLSSEDNADDEVDTRPASFWETS

>sp|P14210|HGF_HUMAN Hepatocyte growth factor OS=Homo sapiens OX=9606 GN=HGF PE=1 SV=2

MWVTKLLPALLLQHVLLHLLLLPIAIPYAEGQRKRRNTIHEFKKSAKTTLIKIDPALKIKTKKVNTADQCANRCTRNKGLPFTCKAFVFDKARKQCLWFPFNSMSSGVKKEFGHEFDLYENKDYIRNCIIGKGRSYKGTVSITKSGIKCQPWSSMIPHEHSFLPSSYRGKDLQENYCRNPRGEEGGPWCFTSNPEVRYEVCDIPQCSEVECMTCNGESYRGLMDHTESGKICQRWDHQTPHRHKFLPERYPDKGFDDNYCRNPDGQPRPWCYTLDPHTRWEYCAIKTCADNTMNDTDVPLETTECIQGQGEGYRGTVNTIWNGIPCQRWDSQYPHEHDMTPENFKCKDLRENYCRNPDGSESPWCFTTDPNIRVGYCSQIPNCDMSHGQDCYRGNGKNYMGNLSQTRSGLTCSMWDKNMEDLHRHIFWEPDASKLNENYCRNPDDDAHGPWCYTGNPLIPWDYCPISRCEGDTTPTIVNLDHPVISCAKTKQLRVVNGIPTRTNIGWMVSLRYRNKHICGGSLIKESWVLTARQCFPSRDLKDYEAWLGIHDVHGRGDEKCKQVLNVSQLVYGPEGSDLVLMKLARPAVLDDFVSTIDLPNYGCTIPEKTSCSVYGWGYTGLINYDGLLRVAHLYIMGNEKCSQHHRGKVTLNESEICAGAEKIGSGPCEGDYGGPLVCEQHKMRMVLGVIVPGRGCAIPNRPGIFVRVAYYAKWIHKIILTYKVPQS

>sp|P24385|CCND1_HUMAN G1/S-specific cyclin-D1 OS=Homo sapiens OX=9606 GN=CCND1 PE=1 SV=1

MEHQLLCCEVETIRRAYPDANLLNDRVLRAMLKAEETCAPSVSYFKCVQKEVLPSMRKIVATWMLEVCEEQKCEEEVFPLAMNYLDRFLSLEPVKKSRLQLLGATCMFVASKMKETIPLTAEKLCIYTDNSIRPEELLQMELLLVNKLKWNLAAMTPHDFIEHFLSKMPEAEENKQIIRKHAQTFVALCATDVKFISNPPSMVAAGSVVAAVQGLNLRSPNNFLSYYRLTRFLSRVIKCDPDCLRACQEQIEALLESSLRQAQQNMDPKAAEEEEEEEEEVDLACTPTDVRDVDI

>sp|P35222|CTNB1_HUMAN Catenin beta-1 OS=Homo sapiens OX=9606 GN=CTNNB1 PE=1 SV=1

MATQADLMELDMAMEPDRKAAVSHWQQQSYLDSGIHSGATTTAPSLSGKGNPEEEDVDTSQVLYEWEQGFSQSFTQEQVADIDGQYAMTRAQRVRAAMFPETLDEGMQIPSTQFDAAHPTNVQRLAEPSQMLKHAVVNLINYQDDAELATRAIPELTKLLNDEDQVVVNKAAVMVHQLSKKEASRHAIMRSPQMVSAIVRTMQNTNDVETARCTAGTLHNLSHHREGLLAIFKSGGIPALVKMLGSPVDSVLFYAITTLHNLLLHQEGAKMAVRLAGGLQKMVALLNKTNVKFLAITTDCLQILAYGNQESKLIILASGGPQALVNIMRTYTYEKLLWTTSRVLKVLSVCSSNKPAIVEAGGMQALGLHLTDPSQRLVQNCLWTLRNLSDAATKQEGMEGLLGTLVQLLGSDDINVVTCAAGILSNLTCNNYKNKMMVCQVGGIEALVRTVLRAGDREDITEPAICALRHLTSRHQEAEMAQNAVRLHYGLPVVVKLLHPPSHWPLIKATVGLIRNLALCPANHAPLREQGAIPRLVQLLVRAHQDTQRRTSMGGTQQQFVEGVRMEEIVEGCTGALHILARDVHNRIVIRGLNTIPLFVQLLYSPIENIQRVAAGVLCELAQDKEAAEAIEAEGATAPLTELLHSRNEGVATYAAAVLFRMSEDKPQDYKKRLSVELTSSLFRTEPMAWNETADLGLDIGAQGEPLGYRQDDPSYRSFHSGGYGQDALGMDPMMEHEMGGHHPGADYPVDGLPDLGHAQDLMDGLPPGDSNQLAWFDTDL

>sp|P51608|MECP2_HUMAN Methyl-CpG-binding protein 2 OS=Homo sapiens OX=9606 GN=MECP2 PE=1 SV=1

MVAGMLGLREEKSEDQDLQGLKDKPLKFKKVKKDKKEEKEGKHEPVQPSAHHSAEPAEAGKAETSEGSGSAPAVPEASASPKQRRSIIRDRGPMYDDPTLPEGWTRKLKQRKSGRSAGKYDVYLINPQGKAFRSKVELIAYFEKVGDTSLDPNDFDFTVTGRGSPSRREQKPPKKPKSPKAPGTGRGRGRPKGSGTTRPKAATSEGVQVKRVLEKSPGKLLVKMPFQTSPGGKAEGGGATTSTQVMVIKRPGRKRKAEADPQAIPKKRGRKPGSVVAAAAAEAKKKAVKESSIRSVQETVLPIKKRKTRETVSIEVKEVVKPLLVSTLGEKSGKGLKTCKSPGRKSKESSPKGRSSSASSPPKKEHHHHHHHSESPKAPVPLLPPLPPPPPEPESSEDPTSPPEPQDLSSSVCKEEKMPRGGSLESDGCPKEPAKTQPAVATAATAAEKYKHRGEGERKDIVSSSMPRPNREEPVDSRTPVTERVS

>sp|P62736|ACTA_HUMAN Actin, aortic smooth muscle OS=Homo sapiens OX=9606 GN=ACTA2 PE=1 SV=1

MCEEEDSTALVCDNGSGLCKAGFAGDDAPRAVFPSIVGRPRHQGVMVGMGQKDSYVGDEAQSKRGILTLKYPIEHGIITNWDDMEKIWHHSFYNELRVAPEEHPTLLTEAPLNPKANREKMTQIMFETFNVPAMYVAIQAVLSLYASGRTTGIVLDSGDGVTHNVPIYEGYALPHAIMRLDLAGRDLTDYLMKILTERGYSFVTTAEREIVRDIKEKLCYVALDFENEMATAASSSSLEKSYELPDGQVITIGNERFRCPETLFQPSFIGMESAGIHETTYNSIMKCDIDIRKDLYANNVLSGGTTMYPGIADRMQKEITALAPSTMKIKIIAPPERKYSVWIGGSILASLSTFQQMWISKQEYDEAGPSIVHRKCF

>sp|Q15672|TWST1_HUMAN Twist-related protein 1 OS=Homo sapiens OX=9606 GN=TWIST1 PE=1 SV=1

MMQDVSSSPVSPADDSLSNSEEEPDRQQPPSGKRGGRKRRSSRRSAGGGAGPGGAAGGGVGGGDEPGSPAQGKRGKKSAGCGGGGGAGGGGGSSSGGGSPQSYEELQTQRVMANVRERQRTQSLNEAFAALRKIIPTLPSDKLSKIQTLKLAARYIDFLYQVLQSDELDSKMASCSYVAHERLSYAFSVWRMEGAWSMSASH

>sp|Q86U44|MTA70_HUMAN N(6)-adenosine-methyltransferase catalytic subunit METTL3 OS=Homo sapiens OX=9606 GN=METTL3 PE=1 SV=2

MSDTWSSIQAHKKQLDSLRERLQRRRKQDSGHLDLRNPEAALSPTFRSDSPVPTAPTSGGPKPSTASAVPELATDPELEKKLLHHLSDLALTLPTDAVSICLAISTPDAPATQDGVESLLQKFAAQELIEVKRGLLQDDAHPTLVTYADHSKLSAMMGAVAEKKGPGEVAGTVTGQKRRAEQDSTTVAAFASSLVSGLNSSASEPAKEPAKKSRKHAASDVDLEIESLLNQQSTKEQQSKKVSQEILELLNTTTAKEQSIVEKFRSRGRAQVQEFCDYGTKEECMKASDADRPCRKLHFRRIINKHTDESLGDCSFLNTCFHMDTCKYVHYEIDACMDSEAPGSKDHTPSQELALTQSVGGDSSADRLFPPQWICCDIRYLDVSILGKFAVVMADPPWDIHMELPYGTLTDDEMRRLNIPVLQDDGFLFLWVTGRAMELGRECLNLWGYERVDEIIWVKTNQLQRIIRTGRTGHWLNHGKEHCLVGVKGNPQGFNQGLDCDVIVAEVRSTSHKPDEIYGMIERLSPGTRKIELFGRPHNVQPNWITLGNQLDGIHLLDPDVVARFKQRYPDGIISKPKNL

>sp|A0PJW8|DAPL1_HUMAN Death-associated protein-like 1 OS=Homo sapiens OX=9606 GN=DAPL1 PE=1 SV=2

MANEVQDLLSPRKGGHPPAVKAGGMRISKKQEIGTLERHTKKTGFEKTSAIANVAKIQTLDALNDALEKLNYKFPATVHMAHQKPTPALEKVVPLKRIYIIQQPRKC

>sp|O14817|TSN4_HUMAN Tetraspanin-4 OS=Homo sapiens OX=9606 GN=TSPAN4 PE=1 SV=1

MARACLQAVKYLMFAFNLLFWLGGCGVLGVGIWLAATQGSFATLSSSFPSLSAANLLIITGAFVMAIGFVGCLGAIKENKCLLLTFFLLLLLVFLLEATIAILFFAYTDKIDRYAQQDLKKGLHLYGTQGNVGLTNAWSIIQTDFRCCGVSNYTDWFEVYNATRVPDSCCLEFSESCGLHAPGTWWKAPCYETVKVWLQENLLAVGIFGLCTALVQILGLTFAMTMYCQVVKADTYCA

>sp|P10589|COT1_HUMAN COUP transcription factor 1 OS=Homo sapiens OX=9606 GN=NR2F1 PE=1 SV=1

MAMVVSSWRDPQDDVAGGNPGGPNPAAQAARGGGGGAGEQQQQAGSGAPHTPQTPGQPGAPATPGTAGDKGQGPPGSGQSQQHIECVVCGDKSSGKHYGQFTCEGCKSFFKRSVRRNLTYTCRANRNCPIDQHHRNQCQYCRLKKCLKVGMRREAVQRGRMPPTQPNPGQYALTNGDPLNGHCYLSGYISLLLRAEPYPTSRYGSQCMQPNNIMGIENICELAARLLFSAVEWARNIPFFPDLQITDQVSLLRLTWSELFVLNAAQCSMPLHVAPLLAAAGLHASPMSADRVVAFMDHIRIFQEQVEKLKALHVDSAEYSCLKAIVLFTSDACGLSDAAHIESLQEKSQCALEEYVRSQYPNQPSRFGKLLLRLPSLRTVSSSVIEQLFFVRLVGKTPIETLIRDMLLSGSSFNWPYMSIQCS

>sp|P32242|OTX1_HUMAN Homeobox protein OTX1 OS=Homo sapiens OX=9606 GN=OTX1 PE=1 SV=1

MMSYLKQPPYGMNGLGLAGPAMDLLHPSVGYPATPRKQRRERTTFTRSQLDVLEALFAKTRYPDIFMREEVALKINLPESRVQVWFKNRRAKCRQQQQSGSGTKSRPAKKKSSPVRESSGSESSGQFTPPAVSSSASSSSSASSSSANPAAAAAAGLGGNPVAAASSLSTPAASSIWSPASISPGSAPASVSVPEPLAAPSNTSCMQRSVAAGAATAAASYPMSYGQGGSYGQGYPTPSSSYFGGVDCSSYLAPMHSHHHPHQLSPMAPSSMAGHHHHHPHAHHPLSQSSGHHHHHHHHHHQGYGGSGLAFNSADCLDYKEPGAAAASSAWKLNFNSPDCLDYKDQASWRFQVL

>sp|P37231|PPARG_HUMAN Peroxisome proliferator-activated receptor gamma OS=Homo sapiens OX=9606 GN=PPARG PE=1 SV=3

MGETLGDSPIDPESDSFTDTLSANISQEMTMVDTEMPFWPTNFGISSVDLSVMEDHSHSFDIKPFTTVDFSSISTPHYEDIPFTRTDPVVADYKYDLKLQEYQSAIKVEPASPPYYSEKTQLYNKPHEEPSNSLMAIECRVCGDKASGFHYGVHACEGCKGFFRRTIRLKLIYDRCDLNCRIHKKSRNKCQYCRFQKCLAVGMSHNAIRFGRMPQAEKEKLLAEISSDIDQLNPESADLRALAKHLYDSYIKSFPLTKAKARAILTGKTTDKSPFVIYDMNSLMMGEDKIKFKHITPLQEQSKEVAIRIFQGCQFRSVEAVQEITEYAKSIPGFVNLDLNDQVTLLKYGVHEIIYTMLASLMNKDGVLISEGQGFMTREFLKSLRKPFGDFMEPKFEFAVKFNALELDDSDLAIFIAVIILSGDRPGLLNVKPIEDIQDNLLQALELQLKLNHPESSQLFAKLLQKMTDLRQIVTEHVQLLQVIKKTETDMSLHPLLQEIYKDLY

>sp|P46937|YAP1_HUMAN Transcriptional coactivator YAP1 OS=Homo sapiens OX=9606 GN=YAP1 PE=1 SV=2

MDPGQQPPPQPAPQGQGQPPSQPPQGQGPPSGPGQPAPAATQAAPQAPPAGHQIVHVRGDSETDLEALFNAVMNPKTANVPQTVPMRLRKLPDSFFKPPEPKSHSRQASTDAGTAGALTPQHVRAHSSPASLQLGAVSPGTLTPTGVVSGPAATPTAQHLRQSSFEIPDDVPLPAGWEMAKTSSGQRYFLNHIDQTTTWQDPRKAMLSQMNVTAPTSPPVQQNMMNSASGPLPDGWEQAMTQDGEIYYINHKNKTTSWLDPRLDPRFAMNQRISQSAPVKQPPPLAPQSPQGGVMGGSNSNQQQQMRLQQLQMEKERLRLKQQELLRQAMRNINPSTANSPKCQELALRSQLPTLEQDGGTQNPVSSPGMSQELRTMTTNSSDPFLNSGTYHSRDESTDSGLSMSSYSVPRTPDDFLNSVDEMDTGDTINQSTLPSQQNRFPDYLEAIPGTNVDLGTLEGDGMNIEGEELMPSLQEALSSDILNDMESVLAATKLDKESFLTWL

>sp|P54851|EMP2_HUMAN Epithelial membrane protein 2 OS=Homo sapiens OX=9606 GN=EMP2 PE=1 SV=1

MLVLLAFIIAFHITSAALLFIATVDNAWWVGDEFFADVWRICTNNTNCTVINDSFQEYSTLQAVQATMILSTILCCIAFFIFVLQLFRLKQGERFVLTSIIQLMSCLCVMIAASIYTDRREDIHDKNAKFYPVTREGSYGYSYILAWVAFACTFISGMMYLILRKRK

>sp|P61586|RHOA_HUMAN Transforming protein RhoA OS=Homo sapiens OX=9606 GN=RHOA PE=1 SV=1

MAAIRKKLVIVGDGACGKTCLLIVFSKDQFPEVYVPTVFENYVADIEVDGKQVELALWDTAGQEDYDRLRPLSYPDTDVILMCFSIDSPDSLENIPEKWTPEVKHFCPNVPIILVGNKKDLRNDEHTRRELAKMKQEPVKPEEGRDMANRIGAFGYMECSAKTKDGVREVFEMATRAALQARRGKKKSGCLVL

>sp|Q13464|ROCK1_HUMAN Rho-associated protein kinase 1 OS=Homo sapiens OX=9606 GN=ROCK1 PE=1 SV=1

MSTGDSFETRFEKMDNLLRDPKSEVNSDCLLDGLDALVYDLDFPALRKNKNIDNFLSRYKDTINKIRDLRMKAEDYEVVKVIGRGAFGEVQLVRHKSTRKVYAMKLLSKFEMIKRSDSAFFWEERDIMAFANSPWVVQLFYAFQDDRYLYMVMEYMPGGDLVNLMSNYDVPEKWARFYTAEVVLALDAIHSMGFIHRDVKPDNMLLDKSGHLKLADFGTCMKMNKEGMVRCDTAVGTPDYISPEVLKSQGGDGYYGRECDWWSVGVFLYEMLVGDTPFYADSLVGTYSKIMNHKNSLTFPDDNDISKEAKNLICAFLTDREVRLGRNGVEEIKRHLFFKNDQWAWETLRDTVAPVVPDLSSDIDTSNFDDLEEDKGEEETFPIPKAFVGNQLPFVGFTYYSNRRYLSSANPNDNRTSSNADKSLQESLQKTIYKLEEQLHNEMQLKDEMEQKCRTSNIKLDKIMKELDEEGNQRRNLESTVSQIEKEKMLLQHRINEYQRKAEQENEKRRNVENEVSTLKDQLEDLKKVSQNSQLANEKLSQLQKQLEEANDLLRTESDTAVRLRKSHTEMSKSISQLESLNRELQERNRILENSKSQTDKDYYQLQAILEAERRDRGHDSEMIGDLQARITSLQEEVKHLKHNLEKVEGERKEAQDMLNHSEKEKNNLEIDLNYKLKSLQQRLEQEVNEHKVTKARLTDKHQSIEEAKSVAMCEMEKKLKEEREAREKAENRVVQIEKQCSMLDVDLKQSQQKLEHLTGNKERMEDEVKNLTLQLEQESNKRLLLQNELKTQAFEADNLKGLEKQMKQEINTLLEAKRLLEFELAQLTKQYRGNEGQMRELQDQLEAEQYFSTLYKTQVKELKEEIEEKNRENLKKIQELQNEKETLATQLDLAETKAESEQLARGLLEEQYFELTQESKKAASRNRQEITDKDHTVSRLEEANSMLTKDIEILRRENEELTEKMKKAEEEYKLEKEEEISNLKAAFEKNINTERTLKTQAVNKLAEIMNRKDFKIDRKKANTQDLRKKEKENRKLQLELNQEREKFNQMVVKHQKELNDMQAQLVEECAHRNELQMQLASKESDIEQLRAKLLDLSDSTSVASFPSADETDGNLPESRIEGWLSVPNRGNIKRYGWKKQYVVVSSKKILFYNDEQDKEQSNPSMVLDIDKLFHVRPVTQGDVYRAETEEIPKIFQILYANEGECRKDVEMEPVQQAEKTNFQNHKGHEFIPTLYHFPANCDACAKPLWHVFKPPPALECRRCHVKCHRDHLDKKEDLICPCKVSYDVTSARDMLLLACSQDEQKKWVTHLVKKIPKNPPSGFVRASPRTLSTRSTANQSFRKVVKNTSGKTS

>sp|Q13625|ASPP2_HUMAN Apoptosis-stimulating of p53 protein 2 OS=Homo sapiens OX=9606 GN=TP53BP2 PE=1 SV=2

MMPMFLTVYLSNNEQHFTEVPVTPETICRDVVDLCKEPGESDCHLAEVWCGSERPVADNERMFDVLQRFGSQRNEVRFFLRHERPPGRDIVSGPRSQDPSLKRNGVKVPGEYRRKENGVNSPRMDLTLAELQEMASRQQQQIEAQQQLLATKEQRLKFLKQQDQRQQQQVAEQEKLKRLKEIAENQEAKLKKVRALKGHVEQKRLSNGKLVEEIEQMNNLFQQKQRELVLAVSKVEELTRQLEMLKNGRIDSHHDNQSAVAELDRLYKELQLRNKLNQEQNAKLQQQRECLNKRNSEVAVMDKRVNELRDRLWKKKAALQQKENLPVSSDGNLPQQAASAPSRVAAVGPYIQSSTMPRMPSRPELLVKPALPDGSLVIQASEGPMKIQTLPNMRSGAASQTKGSKIHPVGPDWSPSNADLFPSQGSASVPQSTGNALDQVDDGEVPLREKEKKVRPFSMFDAVDQSNAPPSFGTLRKNQSSEDILRDAQVANKNVAKVPPPVPTKPKQINLPYFGQTNQPPSDIKPDGSSQQLSTVVPSMGTKPKPAGQQPRVLLSPSIPSVGQDQTLSPGSKQESPPAAAVRPFTPQPSKDTLLPPFRKPQTVAASSIYSMYTQQQAPGKNFQQAVQSALTKTHTRGPHFSSVYGKPVIAAAQNQQQHPENIYSNSQGKPGSPEPETEPVSSVQENHENERIPRPLSPTKLLPFLSNPYRNQSDADLEALRKKLSNAPRPLKKRSSITEPEGPNGPNIQKLLYQRTTIAAMETISVPSYPSKSASVTASSESPVEIQNPYLHVEPEKEVVSLVPESLSPEDVGNASTENSDMPAPSPGLDYEPEGVPDNSPNLQNNPEEPNPEAPHVLDVYLEEYPPYPPPPYPSGEPEGPGEDSVSMRPPEITGQVSLPPGKRTNLRKTGSERIAHGMRVKFNPLALLLDSSLEGEFDLVQRIIYEVDDPSLPNDEGITALHNAVCAGHTEIVKFLVQFGVNVNAADSDGWTPLHCAASCNNVQVCKFLVESGAAVFAMTYSDMQTAADKCEEMEEGYTQCSQFLYGVQEKMGIMNKGVIYALWDYEPQNDDELPMKEGDCMTIIHREDEDEIEWWWARLNDKEGYVPRNLLGLYPRIKPRQRSLA

>sp|P02751|FINC_HUMAN Fibronectin OS=Homo sapiens OX=9606 GN=FN1 PE=1 SV=5

MLRGPGPGLLLLAVQCLGTAVPSTGASKSKRQAQQMVQPQSPVAVSQSKPGCYDNGKHYQINQQWERTYLGNALVCTCYGGSRGFNCESKPEAEETCFDKYTGNTYRVGDTYERPKDSMIWDCTCIGAGRGRISCTIANRCHEGGQSYKIGDTWRRPHETGGYMLECVCLGNGKGEWTCKPIAEKCFDHAAGTSYVVGETWEKPYQGWMMVDCTCLGEGSGRITCTSRNRCNDQDTRTSYRIGDTWSKKDNRGNLLQCICTGNGRGEWKCERHTSVQTTSSGSGPFTDVRAAVYQPQPHPQPPPYGHCVTDSGVVYSVGMQWLKTQGNKQMLCTCLGNGVSCQETAVTQTYGGNSNGEPCVLPFTYNGRTFYSCTTEGRQDGHLWCSTTSNYEQDQKYSFCTDHTVLVQTRGGNSNGALCHFPFLYNNHNYTDCTSEGRRDNMKWCGTTQNYDADQKFGFCPMAAHEEICTTNEGVMYRIGDQWDKQHDMGHMMRCTCVGNGRGEWTCIAYSQLRDQCIVDDITYNVNDTFHKRHEEGHMLNCTCFGQGRGRWKCDPVDQCQDSETGTFYQIGDSWEKYVHGVRYQCYCYGRGIGEWHCQPLQTYPSSSGPVEVFITETPSQPNSHPIQWNAPQPSHISKYILRWRPKNSVGRWKEATIPGHLNSYTIKGLKPGVVYEGQLISIQQYGHQEVTRFDFTTTSTSTPVTSNTVTGETTPFSPLVATSESVTEITASSFVVSWVSASDTVSGFRVEYELSEEGDEPQYLDLPSTATSVNIPDLLPGRKYIVNVYQISEDGEQSLILSTSQTTAPDAPPDTTVDQVDDTSIVVRWSRPQAPITGYRIVYSPSVEGSSTELNLPETANSVTLSDLQPGVQYNITIYAVEENQESTPVVIQQETTGTPRSDTVPSPRDLQFVEVTDVKVTIMWTPPESAVTGYRVDVIPVNLPGEHGQRLPISRNTFAEVTGLSPGVTYYFKVFAVSHGRESKPLTAQQTTKLDAPTNLQFVNETDSTVLVRWTPPRAQITGYRLTVGLTRRGQPRQYNVGPSVSKYPLRNLQPASEYTVSLVAIKGNQESPKATGVFTTLQPGSSIPPYNTEVTETTIVITWTPAPRIGFKLGVRPSQGGEAPREVTSDSGSIVVSGLTPGVEYVYTIQVLRDGQERDAPIVNKVVTPLSPPTNLHLEANPDTGVLTVSWERSTTPDITGYRITTTPTNGQQGNSLEEVVHADQSSCTFDNLSPGLEYNVSVYTVKDDKESVPISDTIIPEVPQLTDLSFVDITDSSIGLRWTPLNSSTIIGYRITVVAAGEGIPIFEDFVDSSVGYYTVTGLEPGIDYDISVITLINGGESAPTTLTQQTAVPPPTDLRFTNIGPDTMRVTWAPPPSIDLTNFLVRYSPVKNEEDVAELSISPSDNAVVLTNLLPGTEYVVSVSSVYEQHESTPLRGRQKTGLDSPTGIDFSDITANSFTVHWIAPRATITGYRIRHHPEHFSGRPREDRVPHSRNSITLTNLTPGTEYVVSIVALNGREESPLLIGQQSTVSDVPRDLEVVAATPTSLLISWDAPAVTVRYYRITYGETGGNSPVQEFTVPGSKSTATISGLKPGVDYTITVYAVTGRGDSPASSKPISINYRTEIDKPSQMQVTDVQDNSISVKWLPSSSPVTGYRVTTTPKNGPGPTKTKTAGPDQTEMTIEGLQPTVEYVVSVYAQNPSGESQPLVQTAVTNIDRPKGLAFTDVDVDSIKIAWESPQGQVSRYRVTYSSPEDGIHELFPAPDGEEDTAELQGLRPGSEYTVSVVALHDDMESQPLIGTQSTAIPAPTDLKFTQVTPTSLSAQWTPPNVQLTGYRVRVTPKEKTGPMKEINLAPDSSSVVVSGLMVATKYEVSVYALKDTLTSRPAQGVVTTLENVSPPRRARVTDATETTITISWRTKTETITGFQVDAVPANGQTPIQRTIKPDVRSYTITGLQPGTDYKIYLYTLNDNARSSPVVIDASTAIDAPSNLRFLATTPNSLLVSWQPPRARITGYIIKYEKPGSPPREVVPRPRPGVTEATITGLEPGTEYTIYVIALKNNQKSEPLIGRKKTDELPQLVTLPHPNLHGPEILDVPSTVQKTPFVTHPGYDTGNGIQLPGTSGQQPSVGQQMIFEEHGFRRTTPPTTATPIRHRPRPYPPNVGEEIQIGHIPREDVDYHLYPHGPGLNPNASTGQEALSQTTISWAPFQDTSEYIISCHPVGTDEEPLQFRVPGTSTSATLTGLTRGATYNVIVEALKDQQRHKVREEVVTVGNSVNEGLNQPTDDSCFDPYTVSHYAVGDEWERMSESGFKLLCQCLGFGSGHFRCDSSRWCHDNGVNYKIGEKWDRQGENGQMMSCTCLGNGKGEFKCDPHEATCYDDGKTYHVGEQWQKEYLGAICSCTCFGGQRGWRCDNCRRPGGEPSPEGTTGQSYNQYSQRYHQRTNTNVNCPIECFMPLDVQADREDSRE

>sp|P04637|P53_HUMAN Cellular tumor antigen p53 OS=Homo sapiens OX=9606 GN=TP53 PE=1 SV=4

MEEPQSDPSVEPPLSQETFSDLWKLLPENNVLSPLPSQAMDDLMLSPDDIEQWFTEDPGPDEAPRMPEAAPPVAPAPAAPTPAAPAPAPSWPLSSSVPSQKTYQGSYGFRLGFLHSGTAKSVTCTYSPALNKMFCQLAKTCPVQLWVDSTPPPGTRVRAMAIYKQSQHMTEVVRRCPHHERCSDSDGLAPPQHLIRVEGNLRVEYLDDRNTFRHSVVVPYEPPEVGSDCTTIHYNYMCNSSCMGGMNRRPILTIITLEDSSGNLLGRNSFEVRVCACPGRDRRTEEENLRKKGEPHHELPPGSTKRALPNNTSSSPQPKKKPLDGEYFTLQIRGRERFEMFRELNEALELKDAQAGKEPGGSRAHSSHLKSKKGQSTSRHKKLMFKTEGPDSD

>sp|P08670|VIME_HUMAN Vimentin OS=Homo sapiens OX=9606 GN=VIM PE=1 SV=4

MSTRSVSSSSYRRMFGGPGTASRPSSSRSYVTTSTRTYSLGSALRPSTSRSLYASSPGGVYATRSSAVRLRSSVPGVRLLQDSVDFSLADAINTEFKNTRTNEKVELQELNDRFANYIDKVRFLEQQNKILLAELEQLKGQGKSRLGDLYEEEMRELRRQVDQLTNDKARVEVERDNLAEDIMRLREKLQEEMLQREEAENTLQSFRQDVDNASLARLDLERKVESLQEEIAFLKKLHEEEIQELQAQIQEQHVQIDVDVSKPDLTAALRDVRQQYESVAAKNLQEAEEWYKSKFADLSEAANRNNDALRQAKQESTEYRRQVQSLTCEVDALKGTNESLERQMREMEENFAVEAANYQDTIGRLQDEIQNMKEEMARHLREYQDLLNVKMALDIEIATYRKLLEGEESRISLPLPNFSSLNLRETNLDSLPLVDTHSKRTLLIKTVETRDGQVINETSQHHDDLE

>sp|P09486|SPRC_HUMAN SPARC OS=Homo sapiens OX=9606 GN=SPARC PE=1 SV=1

MRAWIFFLLCLAGRALAAPQQEALPDETEVVEETVAEVTEVSVGANPVQVEVGEFDDGAEETEEEVVAENPCQNHHCKHGKVCELDENNTPMCVCQDPTSCPAPIGEFEKVCSNDNKTFDSSCHFFATKCTLEGTKKGHKLHLDYIGPCKYIPPCLDSELTEFPLRMRDWLKNVLVTLYERDEDNNLLTEKQKLRVKKIHENEKRLEAGDHPVELLARDFEKNYNMYIFPVHWQFGQLDQHPIDGYLSHTELAPLRAPLIPMEHCTTRFFETCDLDNDKYIALDEWAGCFGIKQKDIDKDLVI

>sp|P12830|CADH1_HUMAN Cadherin-1 OS=Homo sapiens OX=9606 GN=CDH1 PE=1 SV=3

MGPWSRSLSALLLLLQVSSWLCQEPEPCHPGFDAESYTFTVPRRHLERGRVLGRVNFEDCTGRQRTAYFSLDTRFKVGTDGVITVKRPLRFHNPQIHFLVYAWDSTYRKFSTKVTLNTVGHHHRPPPHQASVSGIQAELLTFPNSSPGLRRQKRDWVIPPISCPENEKGPFPKNLVQIKSNKDKEGKVFYSITGQGADTPPVGVFIIERETGWLKVTEPLDRERIATYTLFSHAVSSNGNAVEDPMEILITVTDQNDNKPEFTQEVFKGSVMEGALPGTSVMEVTATDADDDVNTYNAAIAYTILSQDPELPDKNMFTINRNTGVISVVTTGLDRESFPTYTLVVQAADLQGEGLSTTATAVITVTDTNDNPPIFNPTTYKGQVPENEANVVITTLKVTDADAPNTPAWEAVYTILNDDGGQFVVTTNPVNNDGILKTAKGLDFEAKQQYILHVAVTNVVPFEVSLTTSTATVTVDVLDVNEAPIFVPPEKRVEVSEDFGVGQEITSYTAQEPDTFMEQKITYRIWRDTANWLEINPDTGAISTRAELDREDFEHVKNSTYTALIIATDNGSPVATGTGTLLLILSDVNDNAPIPEPRTIFFCERNPKPQVINIIDADLPPNTSPFTAELTHGASANWTIQYNDPTQESIILKPKMALEVGDYKINLKLMDNQNKDQVTTLEVSVCDCEGAAGVCRKAQPVEAGLQIPAILGILGGILALLILILLLLLFLRRRAVVKEPLLPPEDDTRDNVYYYDEEGGGEEDQDFDLSQLHRGLDARPEVTRNDVAPTLMSVPRYLPRPANPDEIGNFIDENLKAADTDPTAPPYDSLLVFDYEGSGSEAASLSSLNSSESDKDQDYDYLNEWGNRFKKLADMYGGGEDD
